# Insights into the Phylogeny, Nodule Function, and Biogeographic Distribution of Microsymbionts Nodulating the Orphan Kersting’s Groundnut [*Macrotyloma geocarpum* (Harms) Marechal & Baudet] in African Soils

**DOI:** 10.1128/AEM.00342-19

**Published:** 2019-05-16

**Authors:** Mustapha Mohammed, Sanjay K. Jaiswal, Felix D. Dakora

**Affiliations:** a Department of Crop Sciences, Tshwane University of Technology, Pretoria, South Africa; b Chemistry Department, Tshwane University of Technology, Pretoria, South Africa; Michigan State University

**Keywords:** *Bradyrhizobium*, legume-rhizobium symbiosis, genetic diversity, photosynthesis, phylogeny

## Abstract

Kersting’s groundnut [Macrotyloma geocarpum (Harms) Marechal & Baudet] is a neglected indigenous African legume adapted to growth in N-deficient soils due to its ability to fix atmospheric N_2_ via symbiosis with rhizobia. Despite its nutritional and medicinal uses, to date there is little information on the phylogeny and functional traits of its microsymbionts, aspects that are much needed for its conservation and improvement. This study explored the morphogenetic diversity, phylogenetic relationships, and N_2_-fixing efficiency of Kersting’s groundnut rhizobial isolates from contrasting environments in Ghana, South Africa, and Mozambique. BOX-PCR fingerprinting revealed high diversity among the rhizobial populations, which was influenced by geographic origin. Of the 164 isolates evaluated, 130 BOX-PCR types were identified at a 70% similarity coefficient, indicating that they were not clones. Soil pH and mineral concentrations were found to influence the distribution of bradyrhizobial populations in African soils. Phylogenetic analysis of 16S rRNA genes and multilocus sequence analysis of protein-coding genes (*atpD*, *glnII*, *gyrB*, and *rpoB*) and symbiotic genes (*nifH* and *nodC*) showed that Kersting’s groundnut is primarily nodulated by members of the genus Bradyrhizobium, which are closely related to Bradyrhizobium vignae 7-2^T^, Bradyrhizobium kavangense 14-3^T^, Bradyrhizobium subterraneum 58-2-1^T^, Bradyrhizobium pachyrhizi PAC48^T^, the type strain of Bradyrhizobium elkanii, and novel groups of Bradyrhizobium species. The bradyrhizobial populations identified exhibited high N_2_ fixation and induced greater nodulation, leaf chlorophyll concentration, and photosynthetic rates in their homologous host than did the 5 mM KNO_3_-fed plants and/or the commercial Bradyrhizobium sp. strain CB756, suggesting that they could be good candidates for inoculant formulations upon field testing.

**IMPORTANCE** Rhizobia play important roles in agroecosystems, where they contribute to improving overall soil health through their symbiotic relationship with legumes. This study explored the microsymbionts nodulating Kersting’s groundnut, a neglected orphan legume. The results revealed the presence of different bradyrhizobial populations with high N_2_-fixing efficiencies as the dominant symbionts of this legume across diverse agroecologies in Africa. Our findings represent a useful contribution to the literature in terms of the community of microsymbionts nodulating a neglected cultivated legume and its potential for elevation as a major food crop. The presence of potentially novel bradyrhizobial symbionts of Kersting’s groundnut found in this study offers an opportunity for future studies to properly describe, characterize, and delineate these isolates functionally and phylogenetically for use in inoculant production to enhance food/nutritional security.

## Abstract (precis)

Rhizobia play important roles in agroecosystems, where they contribute to improving overall soil health through their symbiotic relationship with legumes. This study explored the microsymbionts nodulating Kersting’s groundnut, a neglected orphan legume. The results revealed the presence of different bradyrhizobial populations with high N_2_-fixing efficiencies as the dominant symbionts of this legume across diverse agroecologies in Africa. Our findings represent a useful contribution to the literature in terms of the community of microsymbionts nodulating a neglected cultivated legume and its potential for elevation as a major food crop. The presence of potentially novel bradyrhizobial symbionts of Kersting’s groundnut found in this study offers an opportunity for future studies to properly describe, characterize, and delineate these isolates functionally and phylogenetically for use in inoculant production to enhance food/nutritional security.

## INTRODUCTION

Kersting’s groundnut [Macrotyloma geocarpum (Harms) Marechal & Baudet] is an indigenous African legume which is native to the regions around Togo and central Benin in West Africa (1). This crop is currently cultivated at the subsistence level by older farmers in Ghana and neighboring West African countries (2, 3). It has great potential as a food security crop because of the high protein level (23.1%) and the essential amino acid composition of the grain (4). Kersting’s groundnut is a drought-tolerant legume (5) and is also adapted to growth in N-deficient soils due to its ability to fix atmospheric N_2_ when in symbiosis with soil bacteria called rhizobia. The symbiotic process leads to a high supply of N to the plant and the production of substantial grain yield without external chemical inputs (6, 7). Despite its N_2_-fixing ability, as well as its nutritional and medicinal benefits (2, 8, 9), Kersting’s groundnut is labeled as an orphan crop and is endangered due to neglect by farmers and researchers (10).

As a grain legume adapted to growth under drought and nutrient limitations in African soils (11), Kersting’s groundnut probably harbors novel rhizobia with superior N_2_-fixing traits under those conditions. However, other than the crop being nodulated by Bradyrhizobium sp. strain CB756 under glasshouse conditions (8), there is currently no information on its specific microsymbionts in Africa, its continent of origin. This is despite the fact that studies of rhizobial symbionts of underutilized legumes, such as Kersting’s groundnut, in previously unexplored environments could lead to the identification of compatible and effective rhizobial strains that can support the species’ survival in such locations. For example, similar studies involving the underutilized Bambara groundnut (Vigna subterranea L. Verdc.) revealed the presence of potentially novel microsymbionts responsible for its nodulation in Ghanaian and South African soils (12).

Several studies have reported the presence of high diversity among rhizobial populations responsible for grain legume nodulation in African soils (12–18). Globally, the list of N_2_-fixing rhizobia responsible for legume nodulation consists of over 100 species from 14 bacterial genera originating from diverse legumes and contrasting environments across different continents (19). These sets of information have contributed significantly to the current progress in tapping the benefits of the legume-rhizobium symbiosis for crop production, especially through the use of inoculant formulations with superior N_2_-fixing rhizobial strains (20, 21). However, much remains to be done, as inoculation failures are often encountered when inoculant strains are introduced into new environments (7, 20, 22), thus stressing the need to bioprospect for effective and highly adapted indigenous rhizobial strains for grain legume nodulation in their native environments. Although rhizobial population studies have contributed significantly to our current understanding of the factors influencing their distribution and survival in diverse environments, it is their N_2_-fixing efficacy that is often considered a prerequisite for their selection as strains for inoculant production. Assessing the symbiotic efficiency of rhizobial strains alongside their diversity and phylogeny is therefore a useful approach to tap the agricultural and ecological benefits of the legume-rhizobium symbiosis (15, 21). During the symbiotic process, which commences via intricate molecular dialogue between legumes and their compatible rhizobia, bacteroids in root nodules exchange fixed N for C compounds from photosynthesis (23). As a result, photosynthetic rates and shoot biomass accumulation are often used as measures of N_2_-fixing efficiency in purely symbiotic systems (14).

Despite the nutritional and medicinal value of Kersting’s groundnut, as well as its potential as a food/nutritional security crop, there is currently little information on the microsymbionts associated with its nodulation in African soils. The aim of this study was to provide a detailed insight into the morphogenetic diversity, phylogenetic positions, biogeographic distribution, and symbiotic functioning of indigenous rhizobia associated with Kersting’s groundnut nodulation in Ghana, South Africa, and Mozambique. Although the crop is native to West Africa, this study further explored the potential wider distribution of its microsymbionts outside the cultivation areas (South Africa and Mozambique). The possible effects of host plant and soil factors on the diversity and biogeography of rhizobial isolates were also explored using multivariate analysis.

## RESULTS

### Colony types, morphology, and isolate nodulation ability.

A total of 275 bacterial isolates were obtained from root nodules of Kersting’s groundnut collected from Ghana, South Africa, and Mozambique. Of these isolates, only 164 (60%) could form root nodules on the homologous Kersting’s groundnut host under glasshouse conditions. The nodules induced by the isolates had prominent lenticels and were either brown or black, with the majority located on the crown region of the root. With the exception of isolates TUTMGSA172, TUTMGSA179, and TUTMGSA182 from Klipplaatdrift, South Africa, which were extremely slow in growth (20 to 26 days to first colony appearance), the remaining isolates (98%) were visible on yeast mannitol agar (YMA) plates between 4 and 12 days of incubation at 28°C (see Table S1 in the supplemental material). Colony diameters ranged from <1 mm to 1 mm for 83% of the test isolates, while the remaining 17% had colony diameters ranging from 2 to 4 mm (Table S1).

### Repetitive extragenic palindromic-PCR (BOX-PCR) fingerprints of Kersting’s groundnut isolates.

**FIG 1:**
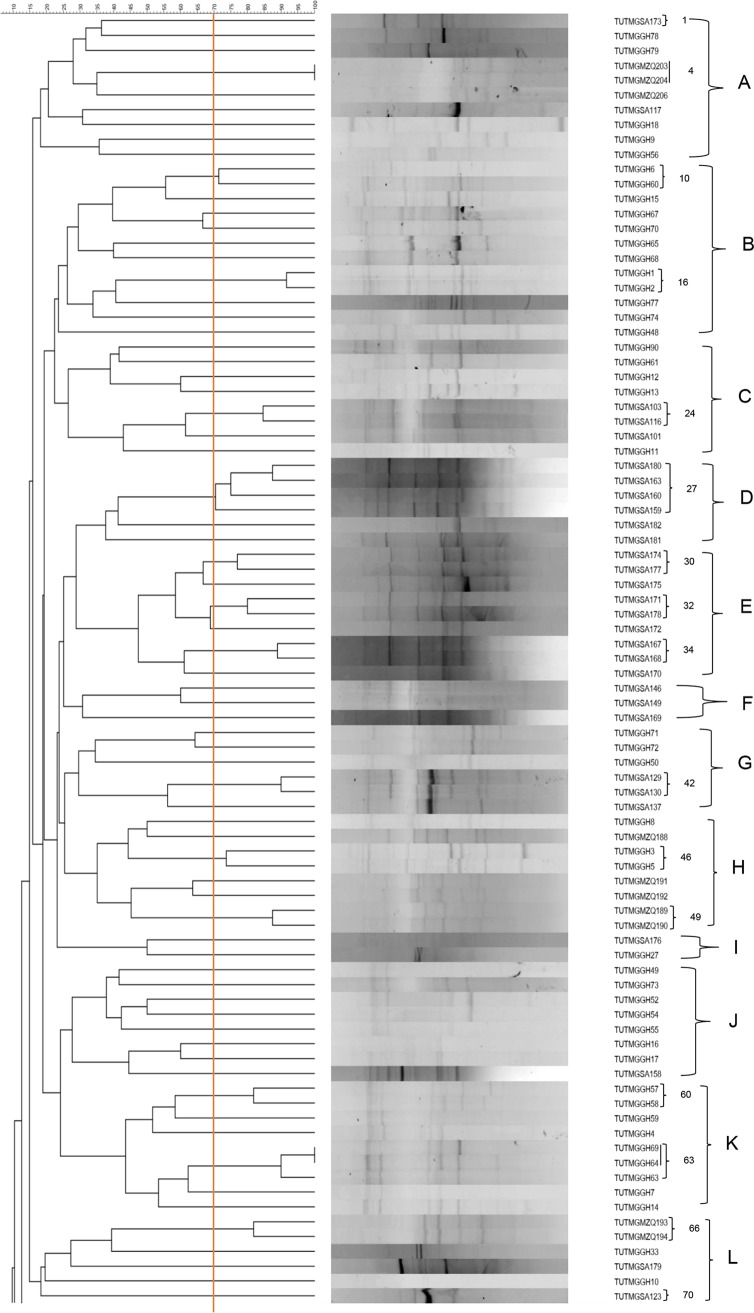

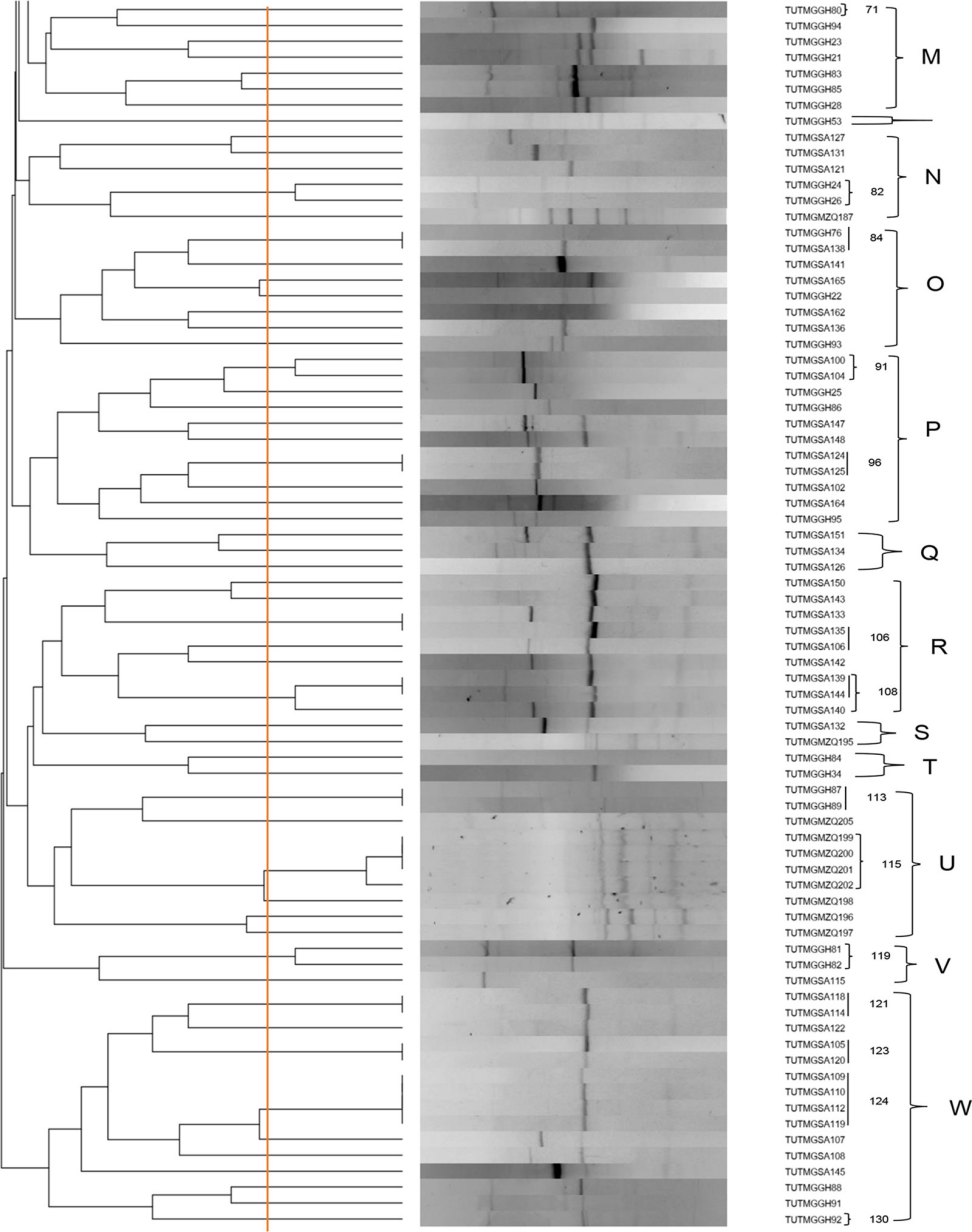
Genomic fingerprints of 164 Kersting’s groundnut rhizobial microsymbionts from Ghana, South Africa, and Mozambique. Larger uppercase letters on the right indicate major clusters. Isolates having distinct BOX-PCR profiles at a cutoff point of 70% similarity (indicated by the vertical red line) are numbered. Where consecutive isolates possess unique PCR profiles, the numbering is skipped and continued at the next group of isolates. Cluster analysis was done with the unweighted pair group method with arithmetic mean (UPGMA) algorithm using the software BioNumerics 7.6.

Genomic fingerprinting of Kersting’s groundnut isolates by BOX-PCR revealed various band sizes ranging from 443 bp to 4,900 bp. The constructed dendrogram grouped the 164 test isolates into 23 major clusters (clusters A to W) comprising 130 BOX-PCR types at a cutoff point of 70% similarity level (Fig. 1). The results of the BOX-PCR analysis showed the presence of country-specific, as well as mixed-country, clustering (Fig. 1). Clusters B, J, K, M, and T comprised only isolates from the locations in Ghana, just as clusters D, E, F, Q, and R contained only isolates from Nelspruit and Klipplaatdrift in South Africa (Fig. 1 and Table S1). In contrast, the remaining 14 major clusters (namely, clusters A, C, G, H, I, J, L, N, O, P, S, U, V, and W) were mixed and contained isolates from the three countries (Fig. 1 and Table S1). Clusters A, L, and N were the most heterogeneous and contained isolates from locations in Ghana, South Africa, and Mozambique (Fig. 1 and Table S1).

**FIG 2:**
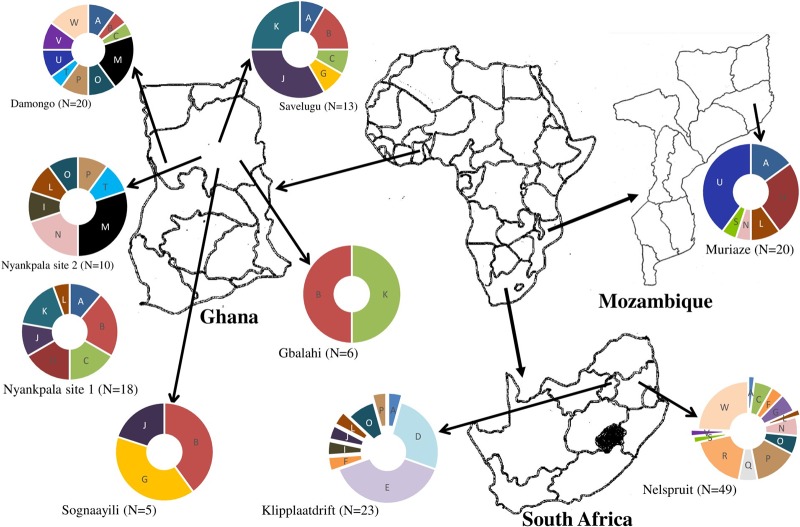
Geographic origin of rhizobial isolates, as well as the distribution of isolates from study locations in different BOX-PCR clusters. For each location, the number of segments indicates the number of BOX-PCR clusters occupied by isolates from that location. Uppercase letters in each segment represent the labels of BOX-PCR clusters as indicated in Fig. 1. The area of each segment is proportional to the number of isolates from a given location occupying that cluster. N, number of different rhizobial isolates.

The results of BOX-PCR analysis showed that many clusters contained isolates from nodules of different landraces (Table S1). Moreover, the diversity of the test isolates was markedly influenced by their geographic origin. When considered on a per-location basis, the Kersting’s groundnut isolates obtained from Nelspruit in South Africa were the most genetically diverse and were present in 13 out of the 23 major BOX-PCR clusters identified (Fig. 2). The isolates obtained from Damongo in Ghana and those from Klipplaatdrift in South Africa were the next group of genetically diverse populations and occupied 10 and 9 clusters, respectively, out of the 23 major clusters (Fig. 2). In contrast, the isolates from Gbalahi and Sognaayili in Ghana were the least diverse populations, probably because of the lower number of isolates obtained from those sites. Nevertheless, despite the lower number of isolates obtained from Nyankpala site 2 (10 isolates) and Nyankpala site 1 (18 isolates), the two populations were similarly diverse and occupied seven clusters each. On the other hand, the 20 isolates from Muriaze in Mozambique were distributed in six clusters (Fig. 2).

Just as the diversity of test isolates varied with geographic origin, it also varied depending on the landrace used as a trap host. The isolates obtained from the Belane Mottled landrace were the least diverse and occupied only 7 out of the 23 major clusters, probably because of their smaller population (Fig. S1). In contrast, the most diverse rhizobial populations were obtained from root nodules of the landrace Funsi, which occupied 18 clusters, followed by those from nodules of landraces Dowie, Sigiri, Puffeun, and then Boli, which were present in 15, 15, 13, and 11 clusters, respectively (Fig. S1). Interestingly, except for the isolates from the Belane Mottled landrace (brown color), which were the least diverse, the other brown-pigmented (Dowie, Funsi, and Sigiri) and black-pigmented (Puffeun) Kersting’s groundnut landraces harbored more diverse rhizobial populations than did the white-seeded Boli (Fig. S1).

### Effect of soil chemical properties on the distribution of bradyrhizobia nodulating Kersting’s groundnut.

**FIG 3:**
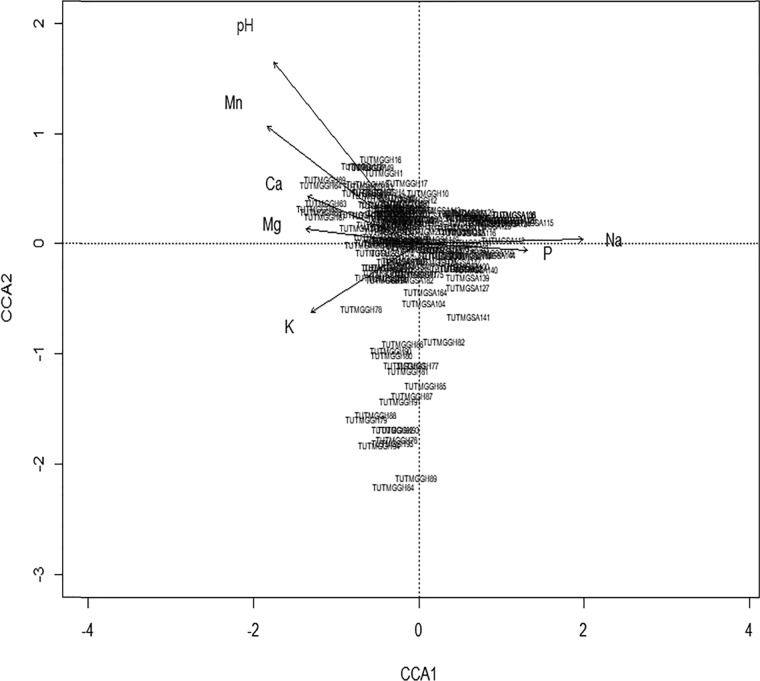
Canonical correspondence analysis (CCA) illustrating the influence of soil chemical properties on the distribution of bradyrhizobia isolated from root nodules of Kersting’s groundnut from contrasting locations in Ghana, South Africa, and Mozambique.

To explore the correlations between the distribution of bradyrhizobial isolates and soil chemical properties of the study locations, a canonical correspondence analysis (CCA) ordination graph was constructed with only those variables (pH and Ca, K, Mg, Mn, Na, and P concentrations) which showed significant influence on bradyrhizobial distribution (*P* ≤ 0.05 at permutation 999) (Fig. 3). The results of the CCA revealed a total mean square contingency coefficient (inertia) of 22.34, of which 53% was explained by both CCA1 and CCA2 axes. Soil Na and P concentrations showed stronger correlations with the positive direction of the first canonical axis (CCA1), while Ca and Mg correlated with the same CCA1 axis but in the negative direction (Fig. 3). Similarly, soil pH and K concentration showed significant correlation in opposite directions with the second canonical axis (CCA2). Furthermore, the concentrations of soil P, Na, Mg, and Ca had a greater influence on the distribution of microsymbionts present on the CCA1 axis, just as pH and soil K concentration showed a greater influence on the isolates on the CCA2 axis. The isolates from Nyankpala were more influenced by soil pH than were isolates from other locations (Fig. 3).

### Phylogeny of Kersting’s groundnut isolates based on 16S rRNA gene sequencing.

**FIG 4:**
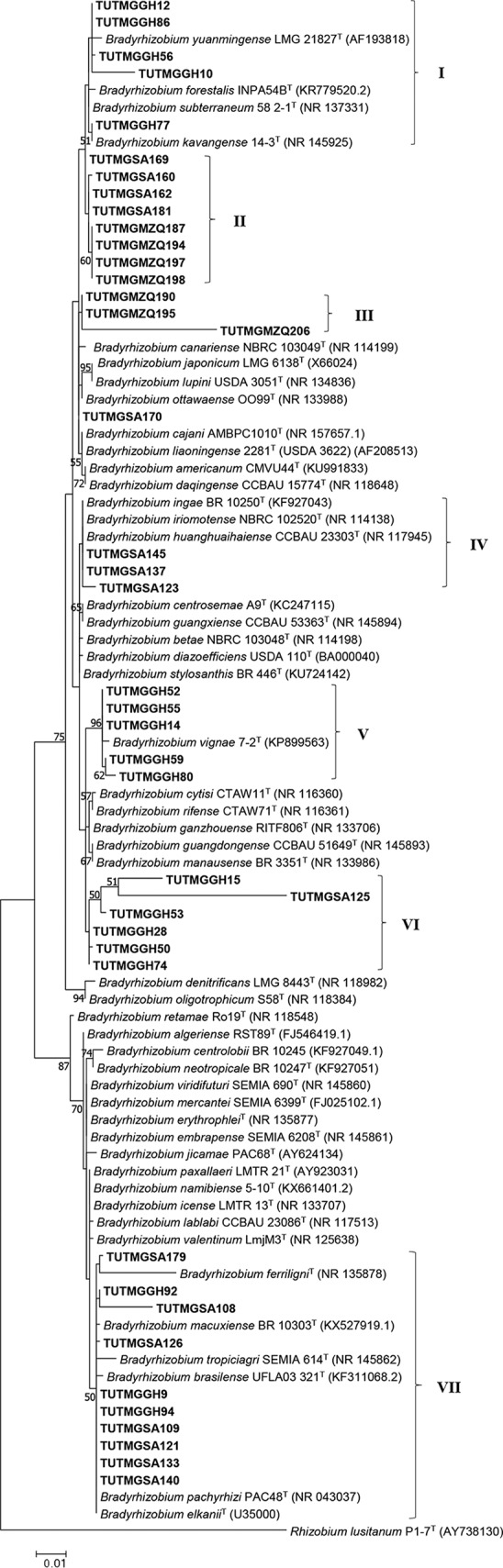
Maximum likelihood molecular phylogenetic analysis of native Kersting’s groundnut isolates from Ghana, South Africa, and Mozambique based on 16S rRNA gene sequences. The analysis involved 90 nucleotide sequences. Roman numerals indicate the labels assigned to each cluster. Accession numbers are listed in parentheses next to the type strain names.

PCR amplification of the 16S rRNA gene yielded bands of approximately 1,500 bp for the 41 representative isolates selected from the different BOX-PCR clusters. Phylogenetic analysis of the 16S rRNA gene assigned the 41 test isolates to seven groups (groups I to VII) within the genus *Bradyrhizobium* (Fig. 4). In group I, isolates TUTMGGH10, TUTMGGH12, TUTMGGH56, TUTMGGH77, and TUTMGGH86 from Ghana shared 98.3 to 100% sequence similarity with B. kavangense 14-3^T^ and B. subterraneum 58 2-1^T^ (Fig. 4). Group II comprised isolates from Mozambique and South Africa (sharing 99.7 to 100% sequence similarity with each other) which stood apart from any reference type strains but shared 99.7 to 99.8% sequence similarity with *B. kavangense* 14-3^T^ (Fig. 4). In group III, isolates TUTMGMZQ190, TUTMGMZQ195, and TUTMGMZQ206 (95.3 to 100% sequence similarity), each obtained from nodules of a different landrace in Mozambique (Table S1), stood separately from any reference type strains but shared 95.1 to 99.7% sequence similarity with Bradyrhizobium cajani AMBPC1010^T^, Bradyrhizobium liaoningense 2281 (USDA 3622)^T^, Bradyrhizobium ottawaense OO99^T^, and *B. kavangense* 14-3^T^, the most closely related reference type strains. Interestingly, each of the isolates in group III occupied a different BOX-PCR cluster (Fig. 1 and 4). In group IV, isolates TUTMGSA123, TUTMGSA137, and TUTMGSA145 (95.3% to 100% sequence similarity) from Nelspruit in South Africa shared close relatedness to Bradyrhizobium huanghuaihaiense, Bradyrhizobium ingae, and Bradyrhizobium iriomotense, with 99.5 to 100% sequence similarity (Fig. 4). Again, the three isolates in group IV each occupied a different BOX-PCR cluster and were also obtained from nodules of different Kersting’s groundnut landraces (Fig. 1 and 4). Although most clusters contained isolates from the same country, isolate TUTMGSA125 from South Africa grouped with other isolates from different locations in Ghana (group VI) (93.3 to 100% sequence similarity) and shared 93.6 to 99.7% sequence similarity with Bradyrhizobium ganzhouense RITF806^T^, *B. huanghuaihaiense* CCBAU 23303^T^, *B. ingae*, and *B. iriomotense* (Fig. 4). Group VII was the largest and similarly comprised isolates from Ghana and South Africa, which shared close relatedness and had 97.8 to 100% sequence similarity with *B. elkanii* and B. pachyrhizi PAC48^T^ (Fig. 4).

### Phylogeny based on housekeeping genes *atpD, glnII, gyrB*, and *rpoB*.

PCR amplification of the *atpD*, *glnII*, *gyrB*, and *rpoB* genes, which code for ATP synthase subunit beta, glutamine synthetase II, DNA gyrase subunit B, and RNA polymerase subunit B, respectively, produced 600-bp, 650-bp, 600-bp, and 550-bp PCR-amplified products. The sequences of *atpD*, *glnII*, *gyrB*, and *rpoB* were obtained for 34, 43, 40, and 43 isolates, respectively. The variations in the number of sequences obtained were due to the poor quality of some sequences. The individual gene-based phylogenies were congruent with each other and with the 16S rRNA-based phylogeny, with few exceptions, and thus placed the test isolates in distinct clusters within the genus *Bradyrhizobium* (Fig. S2 to S5). For example, although isolates TUTMGSA169 and TUTMGSA170 were distantly related in the 16S rRNA phylogeny, the two isolates were tightly grouped in the *atpD* phylogeny (group VI) and shared a close relationship with isolates TUTMGSA181, TUTMGSA160, and TUTMGSA162 in groups V, V, and IV in the *glnII*, *gyrB*, and *rpoB* phylogenies, respectively (Fig. 4 and S2 to S5). Similarly, although isolate TUTMGGH77 showed close relatedness to TUTMGSA160, TUTMGSA162, and TUTMGSA181 in the *atpD* phylogeny, the isolate consistently shared a closer relationship with *B. kavangense* in the *glnII*, *gyrB*, and *rpoB* phylogenies, with 100%, 100%, and 99% sequence similarity, respectively, and 99% bootstrap support (Fig. 4 and S2 to S5).

### Phylogenetic analysis of concatenated sequences.

**FIG 5:**
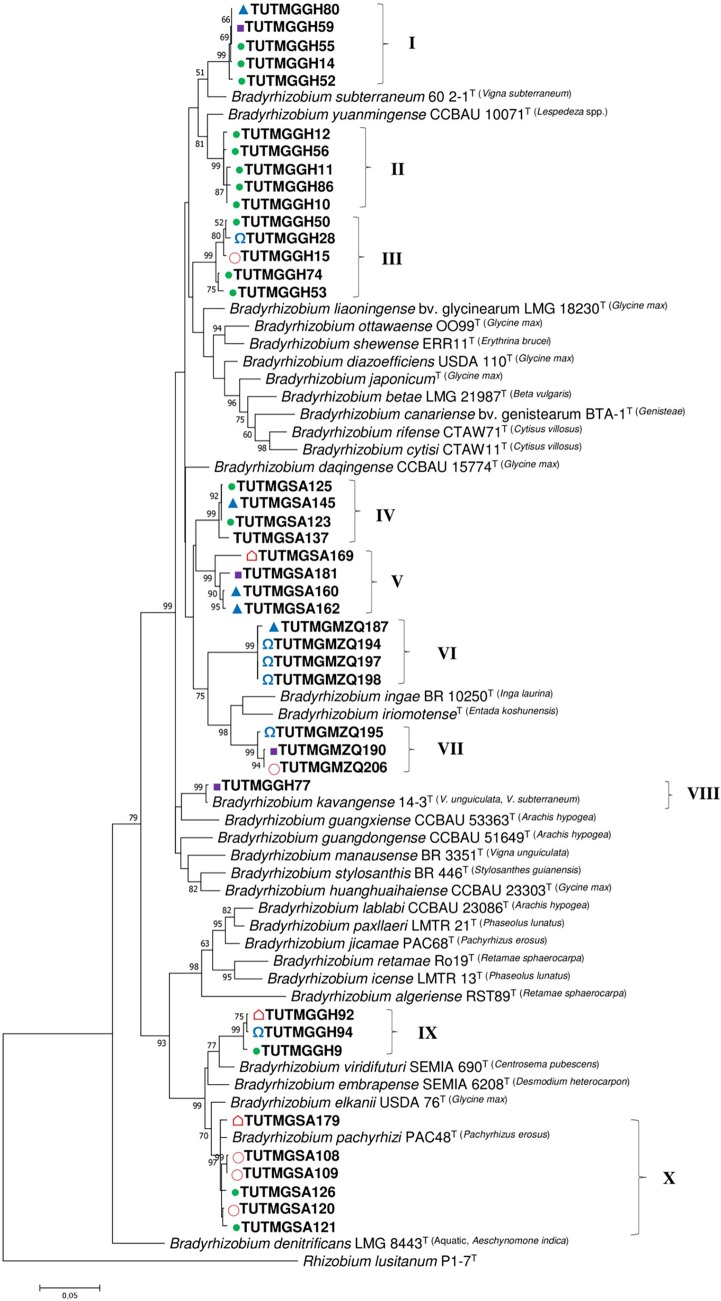
Maximum likelihood phylogenetic analysis of Kersting’s groundnut rhizobial isolates based on concatenated sequences of the *glnII-gyrB-rpoB* genes. The analysis involved 72 nucleotide sequences. Roman numerals indicate the labels assigned to each cluster. For each isolate, the host landrace is indicated by color-coded symbols, as follows: purple square, Puffeun; pink circle, Boli; blue triangle, Dowie; green circle, Funsi; pink pentagon, Sigiri; and blue omega, Belane Mottled. Superscript scientific names in parentheses following the names of type strains indicate their original hosts.

Due to variations in the number of sequences obtained for each gene, two concatenated phylogenetic analyses were carried out based on the sequences of *atpD-glnII-gyrB-rpoB* for 32 isolates (Fig. S6) and *glnII-gyrB-rpoB* for 40 isolates (Fig. 5). The phylogenies based on both concatenated sequences were congruent and successfully refined the discrepancies in the single-gene phylogenies. In both concatenated trees, the test isolates were placed in 10 distinct phylogenetic clusters (clusters I to X) belonging to the genus *Bradyrhizobium* (Fig. 5 and S6). As in the individual gene phylogenies, most of the Kersting’s groundnut isolates were highly divergent from the known *Bradyrhizobium* type strains in the concatenated phylograms. Except for isolates TUTMGSA108, TUTMGSA109, TUTMGSA121, TUTMGSA126, and TUTMGSA179 from South Africa, which grouped with *B. pachyrhizi* PAC48^T^ (98.1 to 99.0% sequence similarity), and isolate TUTMGGH77 from Ghana, which also showed close relatedness to *B. kavangense* 14-3^T^ (99.0% to 99.7% sequence similarity) in the *atpD-glnII-gyrB-rpoB* and *glnII-gyrB-rpoB* phylogenies, the remaining isolates formed distinct clusters with high divergence from the reference type strains (Fig. 5 and S6). Although isolate TUTMGGH52 grouped with *B. vignae* 7-2^T^ in the 16S rRNA, *glnII*, and *rpoB* phylogenies, with 99.6%, 99.1%, and 100% sequence similarity, respectively, the isolate shared a closer relationship with *B. subterraneum* 54 1-1^T^ in the *atpD* and *gyrB* phylogenies, with 96.6% and 96.9% sequence identities, respectively, due to the absence of the *B. vignae* 7-2^T^ strain in the *gyrB* phylogram (Fig. S2 to S5). Consequently, isolate TUTMGGH52 grouped with *B. subterraneum* 54 1-1^T^ in the *atpD-glnII-gyrB-rpoB* phylogeny (group II), with 96.6% sequence identity and 71% bootstrap support (Fig. S6), and together with isolates TUTMGGH14, TUTMGGH55, TUTMGGH59, and TUTMGGH80 formed group I in the *glnII-gyrB-rpoB* phylogeny and showed close relatedness to *B. subterraneum* 54 1-1^T^ (96.6% sequence identity and 99% bootstrap support) (Fig. 5). Isolates TUTMGGH9, TUTMGGH92, and TUTMGGH94 from Ghana (group IX of both concatenated phylograms) showed a high divergence from the reference type strains but shared sequence similarities of 95.7 to 95.8% with *B. pachyrhizi* PAC48^T^ and 96.5 to 96.6% with Bradyrhizobium viridifuturi SEMIA 690^T^, the most closely related type strains (Fig. 5 and S6). Although most isolates from the same country grouped together, they often originated from nodules of different hosts (Fig. 5 and S6).

### Phylogeny based on symbiosis-related (*nifH* and *nodC*) genes.

**FIG 6:**
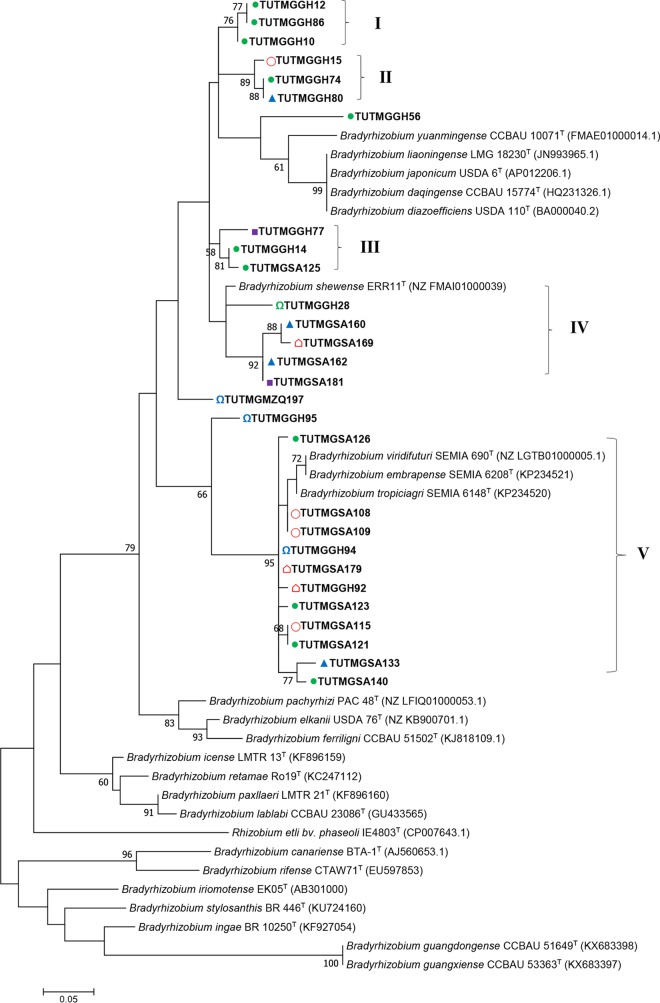
Maximum likelihood molecular phylogenetic analysis of Kersting’s groundnut isolates from Ghana, South Africa, and Mozambique based on sequences of the *nodC* gene. The analysis involved 41 nucleotide sequences. Roman numerals indicate the labels assigned to each cluster. For each isolate, the host landrace is indicated by color-coded symbols, as follows: purple square, Puffeun; pink circle, Boli; blue triangle, Dowie; green circle, Funsi; pink pentagon, Sigiri; and blue omega, Belane Mottled.

PCR amplification of the *nifH* and *nodC* genes yielded 800- and 300-bp band sizes. A total of 35 and 28 sequences were obtained for the *nifH* and *nodC* genes of the test isolates, respectively. The *nifH*- and *nodC*-based phylogenies were congruent with the concatenated gene phylogenies, albeit with few discrepancies (Fig. 5, 6, S6, and S7). For example, although isolates TUTMGGH14, TUTMGGH52, TUTMGGH55, TUTMGGH59, and TUTMGGH80 from Ghana grouped together in all single-gene phylogenies (but were absent in the *atpD* phylogeny, except for TUTMGGH52) and in the concatenated *glnII-gyrB-rpoB* phylogeny, isolate TUTMGGH80 diverged from the other isolates in the *nifH* phylogeny (Fig. S7) and from isolate TUTMGGH14 in the *nodC* phylogeny (Fig. 6). As found with the individual housekeeping gene phylogenies, isolate TUTMGGH14 clustered with TUTMGGH52, TUTMGGH55, and TUTMGGH59 as *B. vignae* in group II of the *nifH* phylogeny (Fig. S7), but it also clustered with isolates TUTMGGH77 and TUTMGSA125 from Ghana and South Africa, respectively, in the *nodC* phylogeny (group III), with 81.7 to 96.7% sequence similarity and 58% bootstrap support (Fig. 6). Furthermore, though isolate TUTMGSA123 belonged to the B. japonicum group in all housekeeping gene and *nifH* gene phylogenies, it clustered with other isolates from Ghana and South Africa from the *B. elkanii* group in the *nodC* phylogeny (90.3 to 100% sequence similarity and 95% bootstrap support in group V) and together with those isolates shared 95.6 to 98.9% sequence similarity with Bradyrhizobium embrapense SEMIA 6208^T^, Bradyrhizobium tropiciagri SEMIA 6148^T^, and Bradyrhizobium viridifuturi SEMIA 690^T^, members of B. viridifuturi sv. tropici in that phylogram (Fig. 6). The remaining isolates formed distinct clusters in the *nodC* phylogeny and showed high divergence from all the known reference type strains or symbiovars (Fig. 6). As with the concatenated trees, all the phylogenetic groups in the *nodC* and *nifH* phylograms contained isolates from different hosts (Fig. 6 and S7).

### Symbiotic efficiency and photosynthetic functioning induced by isolates.

**FIG 7:**
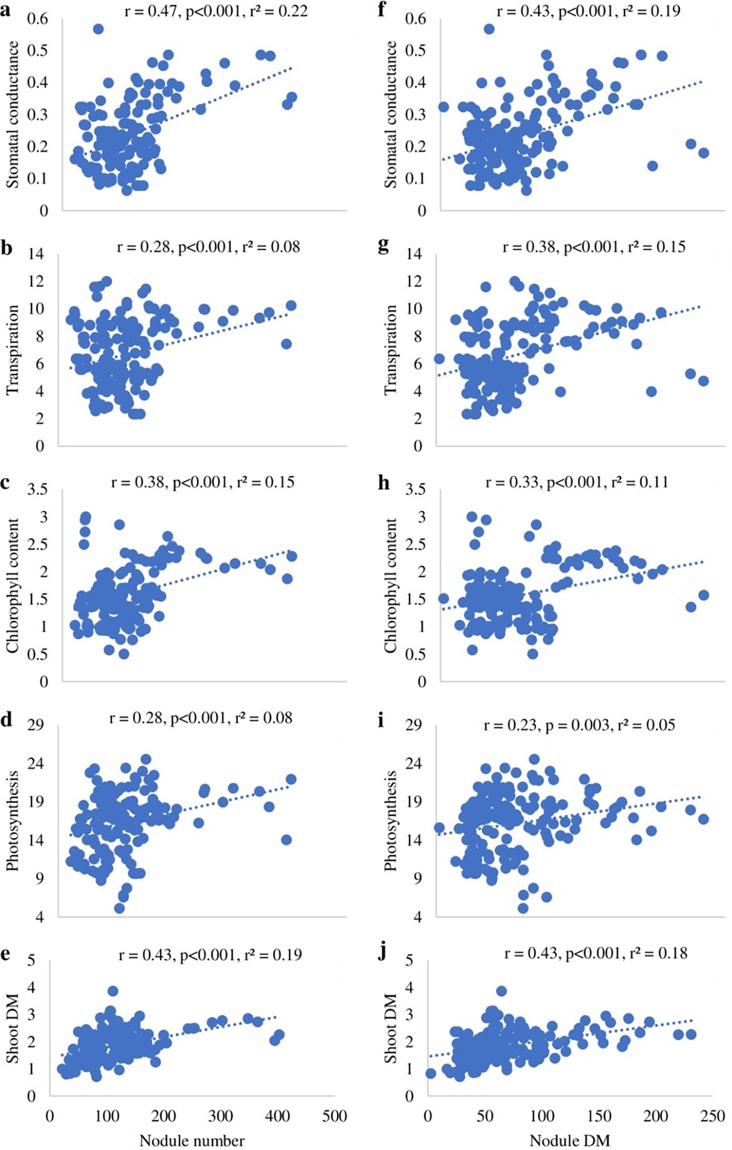
Correlation and regression analysis between nodule number and stomatal conductance (gs) (a), transpiration (E) (b), total chlorophyll content (c), photosynthesis (A) (d), and shoot DM (e), as well as between nodule DM and stomatal conductance (f), transpiration (g), total chlorophyll content (h), photosynthesis (i), and shoot DM (j) induced by Kersting’s groundnut isolates.

A one-way analysis of variance (ANOVA) revealed variable nodulation (nodule number and nodule dry matter [DM]), shoot dry matter accumulation (shoot DM), photosynthetic rates (A), stomatal conductance (gs), plant transpiration (E), leaf chlorophyll content, and relative symbiotic efficiency among representative isolates used in the concatenated *glnII-gyrB-rpoB* phylogenetic analysis (Table 1). The isolates from Mozambique which formed two separate clusters (groups VI and VII) in the *glnII-gyrB-rpoB* phylogram, induced more nodules than did the commercial *Bradyrhizobium* sp. strain CB756 and most other isolates from Ghana and South Africa (Table 1). Isolate TUTMGMZQ206 elicited the highest nodulation (348 ± 23.2 nodules per plant) in Kersting’s groundnut, followed by TUTMGMZQ190, TUTMGMZQ194, TUTMGMZQ198, and TUTMGMZQ187 (303 ± 35.4, 192 ± 19.1, 185 ± 3.3, and 168 ± 15.5 nodules per plant, respectively). Other isolates which also induced higher nodulation than the commercial *Bradyrhizobium* sp. strain CB756 included TUTMGGH52 and TUTMGGH53 from Ghana, TUTMGSA109, TUTMGSA126, TUTMGSA137, TUTMGSA121, and TUTMGSA160 from South Africa, as well as isolate TUTMGMZQ195 from Mozambique (Table 1). In this study, 56% of the test isolates induced nodule numbers similar to that of the commercial strain *Bradyrhizobium* sp. CB756. Increased nodule numbers were generally accompanied by high nodule DM, albeit with few discrepancies due to observed variations in nodule size (Table 1). As a result, there was a significant positive correlation between nodule number and nodule DM induced by the test isolates (*r* = 0.67, *P* < 0.001, *r*^2^ = 0.45). In general, the isolates which elicited greater nodulation also caused an increase in leaf chlorophyll concentrations, stomatal conductance, transpiration rate, photosynthetic rate, and shoot DM (Table 1). These observations were supported by significantly positive correlations obtained when nodule number and nodule DM were each plotted against stomatal conductance, transpiration, photosynthesis, chlorophyll concentration, and shoot DM (Fig. 7). Moreover, there were significantly positive correlations between shoot DM and stomatal conductance (*r* = 0.16, *P* < 0.001, *r*^2^ = 0.03), leaf chlorophyll concentration (*r* = 0.33, *P* < 0.001, *r*^2^ = 0.11), and photosynthetic rates (*r* = 0.33, *P* < 0.001, *r*^2^ = 0.11) induced by the test isolates.

**TABLE 1:** Plant characteristics and RE induced by native rhizobial microsymbionts of Kersting’s groundnut relative to inoculation with *Bradyrhizobium* sp. strain CB756 or with 5 mM KNO_3_ feeding^*a*^

Isolate or group	No. of nodules per plant	Nodule DM (mg) per plant	Shoot DM (g) per plant	A (µmol CO_2_ m^−2^ s^−1^)	gs (mol H_2_O m^−2^ s^−1^)	E (mol H_2_O m^−2^ s^−1^)	Total Chl. concn (mg/g [fresh wt])	RE (%)
TUTMGGH9	80 ± 5.1 LMNOPQ	48.7 ± 7.62 JKLMN	1.93 ± 0.33 CDEFGH	17.4 ± 0.02 EFGHI	0.25 ± 0.00 GHIJKL	5.5 ± 0.01 FGHIJ	1.46 ± 0.04 DEFGH	161 ± 27.8 DEFGHI
TUTMGGH10	80 ± 1.2 LMNOPQ	30.7 ± 0.52 NO	1.74 ± 0.18 FGHIJKL	12.4 ± 0.15 KL	0.17 ± 0.00 LMNOPQ	4.4 ± 0.03 IJKLMN	1.49 ± 0.09 DEFG	145 ± 15.3 FGHIJKL
TUTMGGH11	38 ± 4.0 ST	19.9 ± 6.13 O	1.03 ± 1.14 NO	15.4 ± 0.12 IJ	0.32 ± 0.00 CDEFG	6.3 ± 0.03 EF	1.52 ± 0.03 DEF	86 ± 11.5 N
TUTMGGH12	58 ± 3.0 QRST	33.1 ± 2.77 MNO	1.79 ± 0.22 EFGHIJK	13.9 ± 0.14 JK	0.13 ± 0.01 OPQRST	3.7 ± 0.13 KLMNOP	1.48 ± 0.01 DEFG	150 ± 18.7 EFGHIJK
TUTMGGH14	81 ± 13.7 KLMNOPQ	40.1 ± 2.90 LMNO	1.84 ± 0.15 DEFGHI	11.1 ± 0.38 LMN	0.12 ± 0.02 PQRST	3.1 ± 0.28 NOP	1.39 ± 0.12 DEFGHI	153 ± 12.7 EFGHIJ
TUTMGGH15	73 ± 3.1 OPQR	48.1 ± 0.52 JKLMN	1.36 ± 0.15 HIJKLMNO	9.3 ± 0.25 N	0.09 ± 0.00 RST	2.8 ± 0.11 OP	1.38 ± 0.15 EFGHI	113 ± 12.2 IJKLMN
TUTMGGH28	112 ± 13.4 IJKLMN	90.8 ± 3.86 DE	2.05 ± 0.23 BCDEFG	16.0 ± 1.00 GHI	0.23 ± 0.01 HIJKLM	8.6 ± 0.03 ABC	1.00 ± 0.12 JK	171 ± 19.5 BCDEFGH
TUTMGGH50	111 ± 3.2 IJKLMN	58.9 ± 2.01 IJKL	3.22 ± 0.23 A	12.6 ± 0.10 KL	0.10 ± 0.00 QRST	3.4 ± 0.01 MNOP	1.51 ± 0.18 DEF	268 ± 19.3 A
TUTMGGH52	148 ± 6.6 FGH	40.0 ± 7.83 LMNO	1.96 ± 0.12 CDEFGH	17.7 ± 0.42 DEFGH	0.21 ± 0.01 JKLMN	5.1 ± 0.12 FGHIJK	1.72 ± 0.10 DE	164 ± 10.0 CDEFGHI
TUTMGGH53	135 ± 2.5 FGHIJ	31.9 ± 1.87 MNO	1.65 ± 0.04 FGHIJKLM	9.7 ± 0.00 MN	0.08 ± 0.00 ST	2.3 ± 0.00 P	1.68 ± 0.14 DE	138 ± 3.0 GHIJKLM
TUTMGGH55	101 ± 13.6 JKLMNO	186.3 ± 27.90 A	2.27 ± 0.03 BCDEF	16.1 ± 0.75 GHI	0.17 ± 0.02 LMNOPQR	4.5 ± 0.32 HIJKLMN	1.67 ± 0.13 DE	189 ± 2.2 BCDEFG
TUTMGGH56	77 ± 4.3 NOPQ	33.0 ± 3.98 MNO	2.14 ± 0.10 BCDEFG	17.0 ± 0.02 FGHI	0.24 ± 0.00 HIJKLM	5.7 ± 0.01 EFGHI	1.61 ± 0.13 DE	178 ± 8.5 BCDEFGH
TUTMGGH59	82 ± 7.6 KLMNOPQ	33.2 ± 5.52 MNO	1.10 ± 0.14 MNO	15.2 ± 0.15 IJ	0.23 ± 0.00 IJKLM	5.3 ± 0.01 FGHIJ	0.98 ± 0.14 JK	92 ± 11.6 MN
TUTMGGH74	40 ± 0.6 ST	32.9 ± 2.76 MNO	1.28 ± 0.17 IJKLMNO	16.7 ± 0.03 FGHI	0.27 ± 0.00 FGHIJ	5.6 ± 0.00 FGHIJ	2.79 ± 0.11 A	106 ± 13.8 JKLMN
TUTMGGH77	43 ± 2.0 RST	35.0 ± 5.04 MNO	1.14 ± 0.09 LMNO	11.0 ± 0.39 LMN	0.13 ± 0.01 NOPQRST	8.1 ± 0.30 BCD	1.04 ± 0.07 JK	95 ± 7.5 LMN
TUTMGGH80	83 ± 3.8 KLMNOPQ	68.7 ± 3.52 GHIJ	1.84 ± 0.11 DEFGHI	11.9 ± 0.32 KL	0.19 ± 0.02 KLMNOPQ	9.5 ± 0.71 AB	1.02 ± 0.04 JK	153 ± 9.4 EFGHIJ
TUTMGGH86	28 ± 2.5 T	30.1 ± 5.06 NO	1.00 ± 0.12 O	11.5 ± 0.41 LM	0.17 ± 0.01 MNOPQR	9.3 ± 0.24 AB	1.16 ± 0.12 GHIJ	83 ± 9.9 N
TUTMGGH92	94 ± 11.0 KLMNOPQ	67.6 ± 3.11 GHIJ	1.81 ± 0.15 DEFGHIJ	10.9 ± 0.46 LMN	0.13 ± 0.03 NOPQRS	9.0 ± 1.06 ABC	1.56 ± 0.15 DE	151 ± 12.7 EFGHIJK
TUTMGGH94	113 ± 2.7 HIJKLMN	82.6 ± 4.80 DEFGH	1.95 ± 0.15 CDEFGH	6.6 ± 0.55 O	0.11 ± 0.03 QRST	7.1 ± 1.10 DE	1.19 ± 0.08 FGHIJ	162 ± 12.7 DEFGHI
TUTMGSA108	78 ± 1.3 MNOPQ	42.5 ± 6.22 KLMN	1.65 ± 0.27 FGHIJKLM	18.5 ± 0.35 CDEF	0.20 ± 0.01 JKLMNO	8.7 ± 0.24 ABC	1.18 ± 0.14 FGHIJ	138 ± 22.9 GHIJKLM
TUTMGSA109	160 ± 3.1 DEF	83.1 ± 7.92 DEFGH	1.72 ± 0.10 FGHIJKL	22.5 ± 0.76 A	0.29 ± 0.04 EFGHI	10.0 ± 0.64 A	1.57 ± 0.07 DE	144 ± 8.7 GHIJKLM
TUTMGSA120	64 ± 8.2 PQRS	69.5 ± 6.05 FGHIJ	2.02 ± 0.31 BCDEFG	19.5 ± 1.17 BCDE	0.20 ± 0.03 JKLMNO	7.1 ± 0.63 DE	1.43 ± 0.04 DEFGHI	168 ± 25.5 BCDEFGH
TUTMGSA121	132 ± 13.4 GHIJ	76.4 ± 9.57 EFGHI	2.43 ± 0.17 BCD	17.1 ± 0.73 FGHI	0.14 ± 0.01 NOPQRS	5.4 ± 0.41 FGHIJ	1.16 ± 0.08 GHIJ	202 ± 14.3 BCDE
TUTMGSA123	96 ± 8.0 KLMNOP	53.6 ± 2.16 JKLM	1.70 ± 0.23 FGHIJKLM	21.0 ± 0.83 AB	0.23 ± 0.04 HIJKLM	6.1 ± 0.66 EFG	1.11 ± 0.10 IJK	141 ± 19.4 GHIJKLM
TUTMGSA125	63 ± 7.6 PQRS	35.5 ± 2.82 MNO	1.20 ± 0.06 JKLMNO	19.7 ± 1.54 BCD	0.37 ± 0.07 ABCD	9.2 ± 0.92 ABC	1.40 ± 0.20 DEFGHI	100 ± 4.8 KLMN
TUTMGSA126	142 ± 14.3 FGHI	63.0 ± 1.82 HIJK	2.60 ± 0.14 B	18.1 ± 0.81 CDEFGH	0.17 ± 0.02 LMNOPQR	6.0 ± 0.45 EFG	1.52 ± 0.02 DEF	217 ± 12.0 B
TUTMGSA137	138 ± 6.3 FGHI	84.8 ± 5.01 DEFG	2.13 ± 0.25 BCDEFG	20.9 ± 1.07 AB	0.27 ± 0.04 FGHIJ	8.7 ± 0.91 ABC	1.13 ± 0.08 HIJ	177 ± 20.9 BCDEFGH
TUTMGSA145	92 ± 9.0 KLMNOPQ	90.0 ± 3.42 DEF	1.72 ± 0.24 FGHIJKL	17.3 ± 1.69 EFGHI	0.25 ± 0.03 FGHIJK	9.3 ± 0.67 AB	0.79 ± 0.10 K	144 ± 20.2 GHIJKLM
TUTMGSA160	135 ± 11.1 FGHIJ	51.3 ± 2.66 JKLMN	1.90 ± 0.23 CDEFGHI	20.8 ± 0.58 AB	0.32 ± 0.03 CDEFG	5.9 ± 0.34 EFGH	1.71 ± 0.08 DE	159 ± 19.0 DEFGHIJ
TUTMGSA162	83 ± 7.3 KLMNOPQ	60.2 ± 2.55 IJKLMN	1.68 ± 0.24 FGHIJKLM	20.2 ± 0.16 BC	0.23 ± 0.01 IJKLM	4.8 ± 0.13 GHIJKLM	1.74 ± 0.04 D	140 ± 20.0 GHIJKLM
TUTMGSA169	93 ± 6.8 KLMNOPQ	51.7 ± 2.81 JKLMN	1.71 ± 0.22 FGHIJKLM	17.0 ± 0.14 FGHI	0.21 ± 0.00 JKLMN	4.3 ± 0.07 IJKLMN	1.46 ± 0.05 DEFGH	143 ± 18.6 GHIJKLM
TUTMGSA179	117 ± 1.7 HIJK	62.7 ± 2.56 HIJK	1.56 ± 0.20 GHIJKLMNO	16.0 ± 0.65 HI	0.18 ± 0.03 KLMNOPQ	3.7 ± 0.52 LMNOP	1.70 ± 0.00 DE	130.2 ± 16.8 HIJKLMN
TUTMGSA181	116 ± 15.2 HIJKL	60.4 ± 5.41 IJKL	2.04 ± 0.20 BCDEFG	18.3 ± 0.50 CDEFG	0.19 ± 0.01 JKLMNOP	4.1 ± 0.17 JKLMNO	1.49 ± 0.06 DEFG	170 ± 16.5 BCDEFGH
TUTMGMZQ187	168 ± 15.5 CDE	122.8 ± 13.40 C	1.88 ± 0.09 DEFGHI	18.5 ± 0.23 CDEF	0.39 ± 0.01 ABC	8.8 ± 0.21 ABC	2.29 ± 0.04 BC	156 ± 7.1 DEFGHIJ
TUTMGMZQ190	303 ± 35.4 B	133.1 ± 2.04 C	2.51 ± 0.11 BC	20.9 ± 0.37 AB	0.39 ± 0.02 ABC	10.0 ± 0.08 A	2.23 ± 0.03 BC	209 ± 8.8 BCD
TUTMGMZQ194	192 ± 19.1 C	156.4 ± 5.20 B	2.37 ± 0.24 BCDE	17.1 ± 0.43 FGHI	0.37 ± 0.03 BCDE	9.1 ± 0.30 ABC	2.25 ± 0.04 BC	198 ± 19.6 BCDEF
TUTMGMZQ195	146 ± 10.7 FGHI	121.8 ± 3.73 C	1.95 ± 0.12 CDEFGH	16.3 ± 1.01 FGHI	0.33 ± 0.01 CDEF	7.7 ± 0.17 CD	2.21 ± 0.04 BC	162 ± 10.2 DEFGHI
TUTMGMZQ197	114 ± 5.5 HIJKLM	100.9 ± 6.26 D	2.08 ± 0.25 BCDEFG	17.9 ± 0.52 DEFGH	0.31 ± 0.02 DEFGH	9.1 ± 0.59 ABC	2.27 ± 0.22 BC	174 ± 20.7 BCDEFGH
TUTMGMZQ198	185 ± 3.3 CD	91.9 ± 4.91 DE	1.60 ± 0.13 GHIJKLMN	16.9 ± 0.45 FGHI	0.43 ± 0.03 AB	9.4 ± 0.29 AB	2.44 ± 0.08 B	134 ± 11.1 HIJKLMN
TUTMGMZQ206	348 ± 23.2 A	176.2 ± 7.04 A	2.58 ± 0.18 B	17.9 ± 1.36 DEFGH	0.44 ± 0.04 A	8.9 ± 0.50 ABC	2.03 ± 0.06 C	215 ± 15.3 BC
*Bradyrhizobium* sp. CB756	81 ± 4.3 KLMNOPQ	41.6 ± 1.20 KLMNO	1.68 ± 0.15 FGHIJKLM	18.3 ± 1.07 CDEFG	0.24 ± 0.02 HIJKLM	6.3 ± 0.60 EF	1.70 ± 0.09 DE	140 ± 12.4 GHIJKLM
5 mM KNO_3_ feeding group	NA	NA	1.19 ± 0.03 KLMNO	12.0 ± 0.39 KL	0.14 ± 0.01 NOPQRS	5.1 ± 0.23 FGHIJKL	0.47 ± 0.07 L	NA
Uninoculated (control) group	NA	NA	0.47 ± 0.05 P	1.72 ± 0.10 P	0.06 ± 0.01 T	2.3 ± 0.20 P	0.22 ± 0.04 L	NA

a NA, not applicable; RE, relative symbiotic efficiency. Values (mean ± standard error [SE]; *n* = 4) with dissimilar letters in a column are significantly different at a *P* value of <0.001. F statistics were 34.76 for number of nodules, 37.01 for nodule DM, 7.79 for shoot DM, 40.28 for photosynthesis (A), 17.02 for stomatal conductance (gs), 27.05 for transpiration (E), 25.79 for chlorophyll (Chl.), and 6.17 for RE.RE was calculated as (a/b) × 100 for only the inoculated treatments, where a is the shoot DM of inoculated plants and b is the shoot DM of 5 mM KNO_3_-fed plants.

Of the isolates tested, TUTMGGH50, TUTMGSA121, TUTMGSA126, TUTMGMZQ190, and TUTMGMZQ206 elicited significantly higher relative efficiency (202 to 268%) in terms of shoot biomass accumulation than did the commercial strain CB756 (relative efficiency, 140%). However, except for isolates TUTMGGH11, TUTMGGH59, TUTMGGH77, and TUTMGGH86, which elicited relatively lower nodulation leading to lower efficiency (83.1 to 95.1% relative efficiency), the remaining isolates together with the commercial *Bradyrhizobium* sp. strain CB756 showed high N_2_-fixing efficiency regardless of statistical significance, with relative efficiencies ranging from 100% to 268% (Table 1). In this study, even the relatively lower-performing isolates induced much higher leaf chlorophyll levels than those fed with 5 mM nitrate (Table 1). Of the relatively low-performing isolates, TUTMGGH11 and TUTMGGH86 from Ghana shared close relatedness in the *glnII-gyrB-rpoB* phylogeny, while the remaining isolates were separately grouped in the same phylogram (Fig. 5).

## DISCUSSION

### Kersting’s groundnut is nodulated by genetically diverse bradyrhizobia in African soils.

The identification of diverse rhizobial populations in soils offers an opportunity for the selection of efficient strains for inoculant formulation (13). Moreover, the potential of legume nitrogen fixation for increased crop yields can be enhanced by exploring new regions for novel rhizobia and legume germplasm in support of agriculture from the discovery of elite microsymbionts (24). This study therefore explored the colony characteristics, genetic diversity, and phylogenetic positions of Kersting’s groundnut isolates obtained from diverse agroecologies in Ghana, South Africa, and Mozambique.

In this study, the majority of isolates induced crown nodulation on their homologous Kersting’s groundnut host in the glasshouse, an observation that is usually attributed to low movement of rhizobia in the soil profile (25, 26). The test isolates varied in the number of days to colony appearance and other morphological traits (Table S1), which suggests some diversity. The dendrogram constructed based on BOX-PCR profiles grouped the isolates into 23 major clusters, indicating the presence of genetically diverse rhizobial populations responsible for Kersting’s groundnut nodulation in the studied environments. As with Kersting’s groundnut isolates in this study, high genetic variability has been reported among rhizobial symbionts of several grain legumes, including soybean (27), cowpea (15, 16, 28), Bambara groundnut (12), groundnut (29), and the common bean (17) in African soils, which represents an important biological pool that can be exploited for enhanced symbiosis and increased grain yield through inoculant formulation.

The distribution of Kersting’s groundnut isolates in this study was markedly influenced by the geographic origins of the isolates. The observed tendency for most isolates to show close relatedness in the dendrogram generated from BOX-PCR profiles (Fig. 1) based on common geographic origin suggests the presence of country-specific *Bradyrhizobium* strains responsible for Kersting’s groundnut nodulation in the wider geographic locations studied. The influence of geographic location on the distribution of rhizobia has been previously reported for cowpea isolates from Senegal and Ghana (15, 18, 28). In this study, the isolates from Nelspruit in South Africa were the most diverse, as shown by their presence in 13 out of the 23 major BOX-PCR clusters identified; this is in agreement with earlier studies which also revealed greater diversity among South African bradyrhizobial populations nodulating cowpea and Bambara groundnut than in isolates from Ghana or Botswana (12, 16). Interestingly, although the isolates from Nyankpala sites 1 and 2 in Ghana each occupied seven BOX-PCR clusters, none of these isolates from the two sites occupied the same cluster despite the proximity of their geographic origins (Table S1 and Fig. 2), suggesting that rhizobial diversity can vary greatly between different sites and niches within a geographic location. In this study, the Kersting’s groundnut landraces had no influence on the clustering of the isolates, as most BOX-PCR clusters comprised isolates obtained from nodules of different landraces (Table S1). However, whether the observed higher genetic diversity observed among the nodule occupants of the dark-seeded Kersting’s groundnut landraces in this study was directly or indirectly linked to their greater phenolic compound compositions when compared to that of the white-seeded Boli landrace (30) remains to be explored. Earlier studies have, however, reported a greater abundance of phenolic compounds (involved in signal exchange during the legume-rhizobium symbiosis) in legume seeds with dark seedcoat pigmentation than in those with lighter seedcoat color (31, 32).

### Phylogeny of Kersting’s groundnut rhizobial symbionts native to African soils.

Multilocus sequence analysis in this study revealed the presence of diverse *Bradyrhizobium* species responsible for Kersting’s groundnut nodulation across the contrasting environments in Africa. Phylogenetic analysis assigned the test isolates proximally to *B. vignae* 7-2^T^, *B. subterraneum* 58 2-1^T^, *B. kavangense* 14-3^T^, *B. liaoningense* 2281 (USDA 3622)^T^, Bradyrhizobium yuanmingense LMG 21827^T^, *B. huanghuaihaiense* CCBAU 23303^T^, *B. pachyrhizi* PAC48^T^, and the type strain of *B. elkanii*. However, most of the test isolates were highly divergent from the reference type strains and could represent novel *Bradyrhizobium* species yet to be described. The observed incongruences between the phylogenies of 16S rRNA and protein-coding genes could be attributed to horizontal gene transfer events and/or genetic rearrangement in those genes during evolution (19). However, the fact that isolates TUTMGGG14, TUTMGGH52, TUTMGGH55, TUTMGGH59, and TUTMGGH80 shared a closer relationship with *B. vignae* 7-2^T^ in all the phylogenies, with 99.1 to 99.6% sequence similarity, suggests that *B. vignae* 7-2^T^, a strain originally isolated from root nodules of cowpea and Bambara groundnut in African soils (12, 13), has a broad host range in its nodulation ability. Furthermore, the observed divergence of isolates TUTMGSA160, TUTMGSA162, and TUTMGSA181 from *B. kavangense* 14-3^T^ in the concatenated phylograms (sharing only 94.5 to 95.5% sequence similarity) despite their closeness with the type strain in the 16S rRNA phylogeny (99.7 to 99.8% sequence similarity) is consistent with earlier reports which indicate that high 16S rRNA similarity alone may not yield decisive species delineation (33). According to Helene et al. (33), ≥97% sequence similarity in protein-coding genes can be used as a cutoff for species delineation in bacterial phylogeny. However, except for a few isolates which showed close relatedness to *B. subterraneum* and *B. pachyrhizi* in this study, the majority of the test isolates were distantly related to the reference type strains and could represent novel *Bradyrhizobium* symbionts of Kersting’s groundnut in African soils (Fig. 5 and S6). The presence of high numbers of potentially novel *Bradyrhizobium* species responsible for Kersting’s groundnut nodulation was expected since this study provides a detailed report of the crop’s native symbionts in its cultivation areas (Ghana) and beyond (South Africa and Mozambique).

The phylogenies of *nifH* and *nodC* genes were congruent with each other, as well as with the phylogenies based on individual and concatenated gene sequences, albeit with a few inconsistencies. The incongruences between phylogenies based on symbiotic genes and housekeeping gene loci for some isolates (e.g., TUTMGGH59 and TUTMGGH80) indicate possible horizontal gene transfer and/or genetic recombination events, thus agreeing with earlier reports that symbiosis-related genes such as *nifH* and *nodC* are prone to transfers between bacterial strains (19). However, the observed congruency between phylogenies of symbiotic and housekeeping genes for some isolates in this study could be attributed to common evolutionary histories and/or vertical transfer of those genes to the isolates. Except for isolates in group V of the *nodC* phylogeny, which shared a closer relationship with *B. tropiciagri, B. embrapense*, and *B. viridifuturi* sv. tropici, most isolates in the phylogram (groups I to III) were highly divergent from all reference type strains, indicating that they could belong to novel symbiovars within the *Bradyrhizobium* genus. Although members of *B. viridifuturi* sv. tropici were earlier isolated from nodules of forage legumes in South America (34, 35), a recent study also reported the presence of the symbiovar in root nodules of cowpea grown in African soils (15). Moreover, although isolates in group IV clustered separately in the *nodC* phylogeny, they shared 93.5 to 95.1% sequence similarity with Bradyrhizobium shewense, a strain isolated from Erythrina brucei but with a *nodC* gene similar to that of Bradyrhizobium arachidis isolated from Arachis hypogea (36). It would therefore seem that, like cowpea (another legume of African origin), the Kersting’s groundnut is a promiscuous host which is capable of nodulating with diverse symbiovars within the *Bradyrhizobium* genus. The occurrence of bradyrhizobial isolates from different host landraces in the same phylogenetic clusters of the *nodC* and *nifH* genes suggests that host-symbiont compatibility may be conserved within the species.

### Influence of soil properties on bradyrhizobial distribution.

The distribution of the bradyrhizobial isolates in this study was markedly influenced by the soil chemical properties of the test locations. The South African isolates, which shared close relationships with *B. elkanii* in the phylogenetic analysis, were more influenced by the concentrations of P and Na in the soil. The effect of soil P concentration on the distribution of *B. elkanii* was previously reported by Zhang et al. (37). In another report, the concentration of soil P was also found to alter the diversity of rhizobia in soils (38). In this study, soil K content had a marginal effect on the distribution of isolates from Nyankpala site 1, which were closely related to *B. yuanmingense*, a finding consistent with earlier reports on the effect of soil K on the distribution of *B. yuanmingense* in Chinese soils (39). Recently, Puozaa et al. (12) also found a marked influence of soil mineral nutrients on bradyrhizobial distribution in African soils.

### Symbiotic efficiency and photosynthetic functionality induced by native rhizobial isolates.

Although knowledge of rhizobial biodiversity has contributed to our current understanding of the factors responsible for their survival and distribution, it is their N_2_-fixing efficiency that determines their usefulness for inoculant production aimed at improving the legume-rhizobium symbiosis for increased crop yields (21). In this study, phylogenetically distinct bradyrhizobia responsible for Kersting’s groundnut nodulation were found to show markedly higher symbiotic efficiency than the commercial strain *Bradyrhizobium* sp. CB756 (Table 1). The presence of these potentially novel and high-N_2_-fixing Kersting’s groundnut bradyrhizobia could explain why the crop has survived cultivation in nutrient-depleted soils across West Africa, where landraces were found to exhibit varied responses to *Bradyrhizobium* inoculation (3, 7). As the symbiotic process is sustained by a mutual exchange of N compounds from the rhizobial microsymbiont and photosynthate from the legume host, increased nodulation by test isolates was found to markedly stimulate higher photosynthesis, stomatal conductance, and leaf chlorophyll concentration; consequently, there was increased accumulation of shoot biomass over nitrate feeding. As a result, there were significant positive correlations when nodule number and nodule DM were each plotted against leaf photosynthetic rates, stomatal conductance, plant transpiration, leaf chlorophyll concentration, and shoot biomass (Fig. 7). Thus, increased N_2_ fixation from effective nodulation enhanced RuBisCO and chlorophyll biosynthesis, stimulated greater stomatal conductance, and increased photosynthetic rates, which resulted in better plant growth and biomass accumulation than 5 mM nitrate feeding. Even the relatively low-performing rhizobial isolates induced greater photosynthetic rates and leaf chlorophyll levels, which resulted in 83% to 95% relative efficiency. This finding is consistent with other reports on the superiority of symbiotic N over nitrate feeding in supporting the growth of soybeans (40). Despite the relatively profuse nodulation elicited by isolates from Mozambique which formed distinct clusters in the concatenated trees (Fig. 5), their relative efficiencies were matched by several isolates from Ghana and South Africa which induced moderate nodulation on the homologous host (Table 2), thus agreeing with previous reports that the extent of nodulation is not the sole determinant of symbiotic efficiency (41). Moreover, the presence of highly efficient native rhizobial symbionts of Kersting’s groundnut in the studied countries suggests their wider distribution beyond its current cultivation areas.

**TABLE 2:** Geographic origin of isolates used in this study and the soil chemical properties of the study locations

Country	Region or province	Location (nodule collection method)^*a*^	Geographic coordinates		pH (H_2_O)	% of:		Concn (mg·kg^−1^) of^*b*^:							
			Latitude	Longitude		N	C	B	Ca	Fe	K	Mg	Mn	Na	P
Ghana	Northern	Nyankpala 1 (G)	9.404	−0.982	5.93	0.02	0.4	0.04	250	42	48	55.2	131.4	7	7
		Nyankpala 2 (F)	9.389	−1.006	5.91	0.03	0.40	0.27	212	59.15	68	46.8	79.22	8	6
		Savelugu (G)	9.569	−0.830	5.86	0.02	0.2	0.01	340	44	50	76.8	152.1	4	6
		Gbalahi (G)	9.437	−0.734	6.53	0.03	0.5	0.04	670	34	103	126	142.5	5	7
		Sognaayili (F)	9.447	−0.865	5.59	0.02	0.6	0.01	326	47	51	76.8	156.7	4	7
		Damongo (F)	9.043	−1.814	4.3	0.04	0.31	0.23	226	53.73	90	58.8	54.96	7	10
South Africa	Mpumalanga	Klipplaatdrift (F)	−25.233	29.033	6.0	0.034	0.30	0.23	606	36.80	135	142.8	56.14	11	56
		Nelspruit (F)	−25.474	30.970	4.9	0.029	0.26	0.09	148	38.16	43	45.6	39.90	12	32
Mozambique	Nampula	Muriaze (F)	−15.015	39.495	6.0	0.12	1.3	ND	1212	83.42	134	102.0	216.00	11	21

a Nyankpala site 1, UDS fields; Nyankpala site 2, CSIR-SARI research field. F, rhizobia were trapped by direct planting of Kersting’s bean in the fields; G, rhizobia were trapped in the glasshouse using soils collected from the field. For each study location, soils were randomly sampled from several points on the field and bulked, and subsamples were used for single determination of soil chemical properties.

b ND, not determined.

In conclusion, this study provides a detailed insight into the microsymbiont diversity, biogeographic distribution, and phylogenetic position of rhizobia associated with Kersting’s groundnut nodulation in different African countries. The study further explored the symbiotic efficiency and photosynthetic function elicited by the test bradyrhizobial isolates on homologous Kersting’s groundnut. The results revealed a marked influence of geographic origin and soil mineral nutrient concentrations on the distribution and diversity of potentially novel bradyrhizobial isolates. The observed genetic divergence of most Kersting’s groundnut isolates from *Bradyrhizobium* reference strains in this study suggests that Africa could be a hot spot of bradyrhizobial biodiversity, a finding that compels further studies to be conducted to unravel other novel symbionts responsible for legume nodulation across the continent. Due to their high symbiotic efficiency, the indigenous Kersting’s groundnut isolates in this study represent a useful biological resource that could be tapped for bioinoculant formulations for increased crop yields.

## MATERIALS AND METHODS

### Origin of Kersting’s groundnut nodules used.

The Kersting’s groundnut nodules used in this study were harvested from plants grown at six sites in the Northern Region of Ghana (Nyankpala site 1, Nyankpala site 2, Savelugu, Gbalahi, Sognaayili, and Damongo in the Guinea savanna agroecology), two locations in South Africa (Nelspruit and Klipplaatdrift in the lowveld and middleveld areas of Mpumalanga Province, respectively), and one location (Muriaze) in Mozambique (Table 2 and Fig. 2). The bulk soils from each field were collected, pooled, and analyzed to obtain information on their chemical properties (Table 2). The nodules were either collected from field-grown plants (F) or trapped in the glasshouse (G) using soils collected from the field (Table 2).

### Trapping rhizobia under field conditions.

Six Kersting’s groundnut landraces of various seedcoat colors (namely, Puffeun [black], Boli [white], Dowie [brown mottled], Funsi [brown mottled], Sigiri [brown mottled], and Belane Mottled [brown mottled]) were used to trap rhizobia under field conditions. For this, the seeds of each landrace were surface sterilized in 95% ethanol (3 to 5 minutes), followed by immersion in NaOCl (commercial bleach) for 3 minutes. The seeds were then rinsed 5 times with sterile distilled water by changing the water each time. The sterilized seeds were sown in 3-m by 2-m plots with three replicate plots for each Kersting’s groundnut landrace at Nyankpala 1, Sognaayili, Damongo, Klipplaatdrift, Nelspruit, and Muriaze. Weeds were controlled using a hand hoe. At flowering (55 days after planting), 5 plants were dug out from each plot, separated into shoots and nodulated roots, placed in prelabeled brown paper bags, and transported to the laboratory, where the roots were gently washed in running tap water to remove soil and adhering debris. Nodules were carefully observed and detached with small root segments, dehydrated on silica gel, and stored for bacterial isolation.

### Trapping rhizobia in the glasshouse using soils from the field.

Soils were collected from locations (namely, Nyankpala site 1, Savelugu, and Gbalahi) where field planting was not undertaken and used to trap rhizobia under glasshouse conditions using three phenotypically diverse Kersting’s groundnut landraces (Puffeun, Boli, and Funsi) as hosts. For each landrace, two surface-sterilized seeds were planted in triplicate pots containing sterile (autoclaved) sand. Soil inocula were prepared by suspending 20 g of each soil sample in 1,000 ml of sterile distilled water. The suspension from each location was used to inoculate pots containing seedlings of the different landraces (15). The plants were fed with sterile (autoclaved) N-free nutrient solution (42) and, when necessary, irrigated with sterile distilled water. At flowering, the plants were uprooted and the roots washed with tap water to remove sand and adhering debris. Nodulation was observed, after which the nodules were removed from roots and dehydrated on silica gel for later isolation of root nodule bacteria.

### Isolation of bacteria from root nodules.

Bacterial isolation from root nodules of Kersting’s groundnut was done using the procedure described by Somasegaran and Hoben (43). Healthy and functional nodules (10 to 15 nodules per landrace) were selected from each source for bacterial isolation using yeast mannitol agar (YMA) medium (43). Isolated single colonies were selected from each plate and restreaked onto YMA plates to obtain pure colonies for further characterization. Morphological characteristics, such as colony color, shape, size/diameter, and opacity, were recorded for each isolate. The isolates were named using the prefix for the Tshwane University of Technology (TUT), followed by the scientific name of the crop, Macrotyloma geocarpum (MG), the country of nodule origin (Ghana [GH], South Africa [SA], or Mozambique [MZQ]), and then the serial number during isolation.

### Authentication of bacterial isolates in the glasshouse.

To fulfill Koch’s postulates, single-colony cultures were assessed for their ability to induce root nodules on their homologous Kersting’s groundnut host (using the landrace Funsi) (44). Two surface-sterilized seeds (44) of Kersting’s groundnut were planted in sterile (autoclaved) sand contained in sterile pots. The seedlings were thinned to one plant per pot after germination and grown in a naturally lit glasshouse at an average temperature of 28°C. There were four replicate pots for each isolate. Seven-day-old seedlings were inoculated with 1 ml YMA broth suspension of bacterial culture grown to exponential phase (10^6^ to 10^7^ cells/ml). Uninoculated plants, plants fed 5 mM NO_3_^−1^, and plants inoculated with the commercial strain *Bradyrhizobium* sp. CB756 were used as controls. The inoculated seedlings were supplied with sterile N-free nutrient solution (42) and sterile distilled water in alternation. The plants were harvested at 60 days after planting (DAP) and assessed for nodulation.

### Gas-exchange studies and symbiotic efficiency/effectiveness.

The 40 bacterial isolates selected for multilocus sequence analysis (MLSA) were further evaluated for their N_2_-fixing efficiency. For this, gas-exchange studies were carried out to measure photosynthetic rates (A), stomatal conductance (gs), and leaf transpiration rates (E) on young and fully expanded trifoliate leaves of each replicate plant at 60 DAP using a portable infrared red gas analyzer, version 6.2 (LI-6400XT; LI-COR, Lincoln, NE, USA). The chamber conditions used included photosynthetic flux density of 1,000 μmol m^−2^ s^−1^, reference CO_2_ concentration of 400 μmol mol^−1^, and flow rate of 500 μmol s^−1^. Gas-exchange measurements were carried out between 8:30 a.m. and 12:30 p.m. Leaf chlorophyll concentrations were determined using the same leaves used for gas-exchange measurements. Briefly, chlorophyll was extracted from 6 leaf discs (each with an area of 0.786 cm^2^ and weighing ~18.7 mg) using preheated (65°C) dimethyl sulfoxide for 30 min (45). The absorbance of the extracts at 645 nm and 663 nm was then measured on a Jenway 7300 spectrophotometer. The total chlorophyll concentration was calculated using the equations described by Richardson et al. (45). The plants were then uprooted and assessed for nodulation (nodule number and nodule dry matter) and shoot dry matter (shoot DM) after oven-drying at 60°C for 72 h. The relative efficiency (RE) of isolates was calculated as the shoot biomass of inoculated plants expressed as a percentage of the shoot biomass of the 5 mM KNO_3_-fed Kersting’s groundnut plants (21).

### Extraction of bacterial genomic DNA and BOX-PCR fingerprinting.

The genomic DNA of authenticated rhizobial isolates was extracted using GenElute bacterial genomic DNA kits (Sigma-Aldrich, USA), following the manufacturer’s instructions. DNA integrity was checked on a 0.8% agarose gel stained with ethidium bromide. The bacterial genomic DNA was subjected to repetitive extragenic palindromic-PCR (REP-PCR [BOX-PCR]) using the BOX-A1R primer (46). The final volume of the PCR mixture was 25 μl, and it contained 1 μl (50 to 70 ng/μl) genomic DNA, 12.5 μl MyTaq PCR mastermix (2×) (Bioline USA), 1 μl (10 μM) BOX-A1R primer, and 10.5 ml sterile ultrapure H_2_O. PCR amplification was carried out in a thermal cycler (T100; Bio-Rad, USA) using standard temperature profiles, and the PCR products were subjected to gel electrophoresis (15). Cluster analysis was carried out with the unweighted pair group method with arithmetic mean (UPGMA) algorithm using the trial version of the software BioNumerics 7.6, with kind permission from BioNumerics.

### PCR amplification of the 16S rRNA, protein-coding, and symbiotic genes.

The PCR amplifications of 16S rRNA, protein-coding (*atpD, glnII, gyrB*, and *rpoB*), and symbiotic (*nifH* and *nodC*) genes were each carried out in a 25-μl reaction volume containing 1 μl DNA (50 to 70 ng/μl), 3 μl of MyTaq buffer (5×), 1 μl (10 μM) each of forward and reverse primers of the gene of interest, 0.1 μl *Taq* polymerase (5 U; Bioline, USA), and 18.9 μl double-distilled ultrapure water using standard temperature profiles (15). The amplified gene products were confirmed by gel electrophoresis on a 1.2% agarose gel.

### Sequencing and phylogenetic analysis.

The amplified PCR products were purified using a PCR Cleanup kit (NEB, USA), following the manufacturer’s instructions, and sent to Macrogen (The Netherlands) for sequencing. The quality of sequences was verified using the software BioEdit 7.0.9.0 (47). The BLASTn program was used to identify closely related species in the NCBI database. Pairwise and multiple-sequence alignments were carried out with CLUSTALW, and phylogenetic trees were constructed by means of the maximum likelihood statistical method using MEGA7 software (48). The evolutionary history was inferred by means of the maximum likelihood method based on the Kimura 2-parameter model (49). The robustness of branching was estimated using 1,000 bootstrap replicates (50).

### Statistical analysis.

The influence of soil chemical properties on the distribution of Kersting’s groundnut rhizobial isolates was explored using canonical correspondence analysis (CCA) with vegan (version 2.4-2) (51) in the R software (52). We explored the soil chemical properties of the test locations which influenced the distribution of the Kersting’s groundnut isolates. The general permutation test was used to assess the statistical significance of the ordination axes. The graph analysis is presented only for the soil factors that showed significant contributions. Quantitative data, including nodule number, nodule dry matter, shoot dry matter, photosynthetic rate (A), stomatal conductance (gs), leaf transpiration (E), and total chlorophyll concentration, were subjected to a 1-way ANOVA using the *STATISTICA* software version 10.0 (StatSoft). The Duncan multiple-range test was employed to separate the means that showed significant differences at a *P* value of ≤0.05.

### Data availability.

The sequences obtained were deposited in the NCBI GenBank database with the accession numbers MK183839 to MK183879 for the 16S rRNA gene, MK183880 to MK183913 for *atpD*, MK183914 to MK183953 for *gyrB*, MK183954 to MK183996 for *rpoB*, MK184040 to MK184078 for *nifH*, MK184079 to MK184106 for *nodC*, and MK184107 to MK184149 for *glnII*.

## Supplementary Material

Supplemental file 1
