# Supplementary material for: Insights into the Phylogeny, Nodule Function, and Biogeographic Distribution of Microsymbionts Nodulating the Orphan Kersting’s Groundnut [Macrotyloma geocarpum (Harms) Marechal & Baudet] in African Soils
Source: Appl Environ Microbiol. 2019 May 16;85(11):e00342-19. doi: 10.1128/AEM.00342-19 (PMC6532025; doi:10.1128/AEM.00342-19)
Supplement: Supplemental file 1 [file AEM.00342-19-s0001.pdf]

Table S1 Geographic origin (country and location), host landrace, and the morphological description (size, shape, colour and appearance/opacity) of indigenous rhizobial isolates/colonies used in this study. Growth refers to the number of days taken for colonies to appear on yeast mannitol agar plates and size indicates colony diameter. [1] Nyankpala site 1 (UDS fields), [2] Nyankpala site 2 (CSIR-SARI research field). Cluster (≥ 70% similarity) refers to Box-PCR profiles in Fig. 1.

Isolate	Country of Origin	location	Cluster (≥70% similarity)	Major Cluster	Landrace	Growth (days)	Size (mm)	Shape	Colour	Opacity
TUTMGSA173	South Africa	Klipplaatdrift	1		Dowie	7	<1	Round	Watery	Translucent
TUTMGH78	Ghana	Damongo	2		Puffeun	6	<1	Round	Cream	Opaque
TUTMGGH79	Ghana	Damongo	3		Puffeun	7	<1	Round	Cream	Opaque
TUTMGMZQ203	Mozambique	Muriaze	4		Boli	6	2	Round	Watery	Opaque
TUTMGMZQ204	Mozambique	Muriaze	4	A	Boli	6	2	Round	Cream	Opaque
TUTMGMZQ206	Mozambique	Muriaze	5		Boli	9	2	Round	Cream	Opaque
TUTMGSA117	South Africa	Nelspruit	6		Boli	6	1	Round	Watery	Translucent
TUTMGGH18	Ghana	Nyankpala[1]	7		Boli	6	1	Round	Watery	Translucent
TUTMGGH9	Ghana	Nyankpala[1]	8		Funsi	6	2	Oval	Watery	Translucent
TUTMGGH56	Ghana	Savelugu	9		Funsi	6	<1	Round	Watery	Translucent
TUTMGGH6	Ghana	Nyankpala[1]	10		Boli	6	3	Round	Watery	Translucent
TUTMGGH60	Ghana	Savelugu	10		Puffeun	6	<1	Round	Cream	Opaque
TUTMGGH15	Ghana	Nyankpala[1]	11		Boli	6	<1	Round	Watery	Translucent
TUTMGGH67	Ghana	Gbalahi	12		Funsi	6	<1	Round	Cream	Opaque
TUTMGGH70	Ghana	Sognaayili	13		Funsi	6	5	Convex	Watery	Translucent
TUTMGGH65	Ghana	Gbalahi	14	B	Funsi	6	<1	Round	Watery	Translucent
TUTMGGH68	Ghana	Gbalahi	15		Funsi	6	<1	Round	Watery	Translucent
TUTMGGH1	Ghana	Nyankpala[1]	16		Puffeun	6	4	Convex	Watery	Translucent
TUTMGGH2	Ghana	Nyankpala[1]	16		Puffeun	6	3	Round	Watery	Translucent
TUTMGGH77	Ghana	Damongo	17		Puffeun	9	<1	Round	Watery	Translucent
TUTMGGH74	Ghana	Sognaayili	18		Funsi	6	2	Round	Cream	Opaque
TUTMGGH48	Ghana	Savelugu	19		Funsi	4	2	Round	Watery	Translucent
TUTMGGH90	Ghana	Damongo	20		Sigiri	7	<1	Round	Cream	Opaque
TUTMGGH61	Ghana	Savelugu	21		Puffeun	6	<1	Round	Watery	Translucent
TUTMGGH12	Ghana	Nyankpala[1]	22		Funsi	6	2	Round	Yellow	Opaque
TUTMGGH13	Ghana	Nyankpala[1]	23	C	Funsi	6	4	Round	Yellow	Opaque
TUTMGSA103	South Africa	Nelspruit	24		Puffeun	6	1	Round	Watery	Translucent
TUTMGSA116	South Africa	Nelspruit	24		Boli	6	2	Round	Watery	Translucent
TUTMGSA101	South Africa	Nelspruit	25		Puffeun	9	2	Convex	Watery	Translucent
TUTMGGH11	Ghana	Nyankpala[1]	26		Funsi	6	<1	Round	Brown	Opaque
TUTMGSA159	South Africa	Klipplaatdrift	27		Funsi	7	1	Round	Cream	Opaque
TUTMGSA160	South Africa	Klipplaatdrift	27		Dowie	9	<1	Round	Watery	Translucent
TUTMGSA163	South Africa	Klipplaatdrift	27	D	Sigiri	7	1	Round	Cream	Opaque
TUTMGSA180	South Africa	Klipplaatdrift	27		Dowie	7	<1	Round	Watery	Translucent
TUTMGSA182	South Africa	Klipplaatdrift	28		Sigiri	20	<1	Round	Watery	Translucent
TUTMGSA181	South Africa	Klipplaatdrift	29		Puffeun	9	1	Round	Cream	Opaque

Table S1
continued

Isolate	Country of Origin	location	Cluster (≥70% similarity)	Major Cluster	Landrace	Growth (days)	Size (mm)	Shape	Colour	Opacity
TUTMGSA174	South Africa	Klipplaatdrift	30		Dowie	9	<1	Round	Watery	Translucent
TUTMGSA177	South Africa	Klipplaatdrift	30		Dowie	7	<1	Round	Watery	Translucent
TUTMGSA175	South Africa	Klipplaatdrift	31		Sigiri	9	<1	Round	Watery	Translucent
TUTMGSA171	South Africa	Klipplaatdrift	32		Sigiri	9	1	Round	Cream	Opaque
TUTMGSA178	South Africa	Klipplaatdrift	32	E	Dowie	9	1	Round	Watery	Translucent
TUTMGSA172	South Africa	Klipplaatdrift	33		Belane M	25	1	Round	Cream	Opaque
TUTMGSA167	South Africa	Klipplaatdrift	34		Puffeun	9	<1	Round	Watery	Translucent
TUTMGSA168	South Africa	Klipplaatdrift	34		Boli	5	<1	Round	Watery	Translucent
TUTMGSA170	South Africa	Klipplaatdrift	35		Sigiri	9	3	Convex	Cream	Opaque
TUTMGSA146	South Africa	Nelspruit	36		Dowie	6	3	Oval	Watery	Translucent
TUTMGSA149	South Africa	Nelspruit	37	F	Dowie	6	1	Round	Watery	Translucent
TUTMGSA169	South Africa	Klipplaatdrift	38		Sigiri	9	1	Round	Milky	Opaque
TUTMGGH71	Ghana	Sognaayili	39		Funsi	7	2	Convex	Watery	Translucent
TUTMGGH72	Ghana	Sognaayili	40		Puffeun	6	2	Round	Cream	Opaque
TUTMGGH50	Ghana	Savelugu	41	G	Funsi	6	<1	Round	Cream	Opaque
TUTMGSA129	South Africa	Nelspruit	42		Boli	3	1	Round	Brown	Opaque
TUTMGSA130	South Africa	Nelspruit	42		Funsi	5	3	Round	Watery	Translucent
TUTMGSA137	South Africa	Nelspruit	43		Sigiri	6	2	Oval	Watery	Translucent
TUTMGGH8	Ghana	Nyankpala[1]	44		Funsi	7	<1	Round	Cream	Opaque
TUTMGMZQ188	Mozambique	Muriaze	45		Dowie	6	<1	Round	Watery	Translucent
TUTMGGH3	Ghana	Nyankpala[1]	46		Puffeun	6	<1	Round	Cream	Opaque
TUTMGGH5	Ghana	Nyankpala[1]	46	H	Puffeun	6	1	Round	Watery	Translucent
TUTMGMZQ191	Mozambique	Muriaze	47		Funsi	6	3	Round	Cream	Opaque
TUTMGMZQ192	Mozambique	Muriaze	48		Belane M	4	4	Oval	Cream	Opaque
TUTMGMZQ189	Mozambique	Muriaze	49		Puffeun	6	3	Convex	Watery	Translucent
TUTMGMZQ190	Mozambique	Muriaze	49		Puffeun	6	3	Round	Watery	Translucent
TUTMGSA176	South Africa	Klipplaatdrift	50	I	Sigiri	9	<1	Round	Cream	Opaque
TUTMGGH27	Ghana	Nyankpala[2]	51		Funsi	5	1	Round	Watery	Translucent
TUTMGGH49	Ghana	Savelugu	52		Funsi	6	1	Round	Cream	Opaque
TUTMGGH73	Ghana	Sognaayili	53		Sigiri	9	<1	Round	Cream	Opaque
TUTMGGH52	Ghana	Savelugu	54		Funsi	6	<1	Round	Cream	Opaque
TUTMGGH54	Ghana	Savelugu	55	J	Funsi	6	<1	Round	Cream	Opaque
TUTMGGH55	Ghana	Savelugu	56		Funsi	6	1	Round	Cream	Opaque
TUTMGGH16	Ghana	Nyankpala[1]	57		Boli	6	4	Round	Watery	Translucent
TUTMGGH17	Ghana	Nyankpala[1]	58		Boli	6	<1	Round	Watery	Translucent
TUTMGSA158	South Africa	Klipplaatdrift	59		Dowie	9	2	Round	Cream	Opaque
TUTMGGH57	Ghana	Savelugu	60		Funsi	6	1	Round	Cream	Opaque
TUTMGGH58	Ghana	Savelugu	60		Puffeun	7	<1	Round	Cream	Opaque
TUTMGGH59	Ghana	Savelugu	61		Puffeun	6	<1	Round	Cream	Opaque
TUTMGGH4	Ghana	Nyankpala[1]	62		Puffeun	6	<1	Round	Cream	Opaque
TUTMGGH63	Ghana	Gbalahi	63	K	Puffeun	4	<1	Round	Cream	Opaque
TUTMGGH64	Ghana	Gbalahi	63		Puffeun	6	<1	Round	Cream	Opaque
TUTMGGH69	Ghana	Gbalahi	63		Funsi	6	1	Round	Cream	Opaque
TUTMGGH7	Ghana	Nyankpala[1]	64		Funsi	6	<1	Round	Cream	Opaque
TUTMGGH14	Ghana	Nyankpala[1]	65		Funsi	6	<1	Round	Cream	Opaque

Table S1
continued

Isolate	Country of Origin	location	Cluster (≥70% similarity)	Major Cluster	Landrace	Growth (days)	Size (mm)	Shape	Colour	Opacity
TUTMGMZQ193	Mozambique	Muriaze	66	L	Belane M	6	1	Round	Cream	Opaque
TUTMGMZQ194	Mozambique	Muriaze	66		Belane M	9	<1	Round	Cream	Opaque
TUTMGGH33	Ghana	Nyankpala[2]	67		Sigiri	8	1	Round	Watery	Translucent
TUTMGSA179	South Africa	Klipplaatdrift	68		Sigiri	26	<1	Round	Cream	Opaque
TUTMGGH10	Ghana	Nyankpala[1]	69		Funsi	6	1	Round	Watery	Translucent
TUTMGSA123	South Africa	Nelspruit	70		Funsi	6	1	Convex	Watery	Translucent
TUTMGGH80	Ghana	Damongo	71	M	Dowie	6	<1	Round	Watery	Translucent
TUTMGGH94	Ghana	Damongo	72		Belane M	6	<1	Round	Watery	Translucent
TUTMGGH23	Ghana	Nyankpala[2]	73		Boli	2	<1	Round	Watery	Translucent
TUTMGGH21	Ghana	Nyankpala[2]	74		Puffeun	8	<1	Round	Watery	Translucent
TUTMGGH83	Ghana	Damongo	75		Funsi	6	<1	Round	Watery	Translucent
TUTMGGH85	Ghana	Damongo	76		Funsi	9	<1	Round	Cream	Opaque
TUTMGGH28	Ghana	Nyankpala[2]	77		Belane M	9	<1	Round	Watery	Translucent
TUTMGGH53	Ghana	Savelugu	78	N	Funsi	6	<1	Round	Watery	Translucent
TUTMGSA127	South Africa	Nelspruit	79		Funsi	6	<1	Round	Cream	Opaque
TUTMGSA131	South Africa	Nelspruit	80		Funsi	5	1	Round	Watery	Translucent
TUTMGSA121	South Africa	Nelspruit	81		Funsi	6	1	Round	Watery	Translucent
TUTMGGH24	Ghana	Nyankpala[2]	82		Boli	7	<1	Round	Cream	Opaque
TUTMGGH26	Ghana	Nyankpala[2]	82		Funsi	7	<1	Round	Cream	Opaque
TUTMGMZQ187	Mozambique	Muriaze	83		Dowie	6	<1	Round	Watery	Translucent
TUTMGGH76	Ghana	Damongo	84	O	Puffeun	12	1	Round	Watery	Translucent
TUTMGSA138	South Africa	Nelspruit	84		Sigiri	6	2	Oval	Watery	Translucent
TUTMGSA141	South Africa	Nelspruit	85		Sigiri	8	<1	Round	Watery	Translucent
TUTMGSA165	South Africa	Klipplaatdrift	86		Dowie	7	<1	Round	Watery	Translucent
TUTMGGH22	Ghana	Nyankpala[2]	87		Puffeun	6	<1	Round	Watery	Translucent
TUTMGSA162	South Africa	Klipplaatdrift	88		Dowie	7	1	Round	Watery	Translucent
TUTMGSA136	South Africa	Nelspruit	89		Sigiri	6	2	Round	Watery	Translucent
TUTMGGH93	Ghana	Damongo	90		Sigiri	9	<1	Round	Watery	Translucent
TUTMGSA100	South Africa	Nelspruit	91	P	Puffeun	9	1	Round	Watery	Translucent
TUTMGSA104	South Africa	Nelspruit	91		Puffeun	6	2	Round	Watery	Translucent
TUTMGGH25	Ghana	Nyankpala[2]	92		Funsi	7	1	Round	Cream	Opaque
TUTMGGH86	Ghana	Damongo	93		Funsi	6	<1	Round	Brown	Opaque
TUTMGSA147	South Africa	Nelspruit	94		Dowie	6	3	Round	Watery	Translucent
TUTMGSA148	South Africa	Nelspruit	95		Sigiri	6	3	Irregular	Watery	Translucent
TUTMGSA124	South Africa	Nelspruit	96		Funsi	6	3	Round	Watery	Translucent
TUTMGSA125	South Africa	Nelspruit	96		Funsi	6	3	Round	Watery	Translucent
TUTMGSA102	South Africa	Nelspruit	97		Puffeun	8	<1	Round	Watery	Translucent
TUTMGSA164	South Africa	Klipplaatdrift	98		Puffeun	7	1	Round	Watery	Translucent
TUTMGGH95	Ghana	Damongo	99		Belane M	2	4	Round	Cream	Opaque

Table S1
continued

Isolate	Country of Origin	location	Cluster (≥70% similarity)	Major Cluster	Landrace	Growth (days)	Size (mm)	Shape	Colour	Opacity
TUTMGSA151	South Africa	Nelspruit	100		Dowie	6	1	Round	Watery	Translucent
TUTMGSA134	South Africa	Nelspruit	101	Q	Sigiri	6	2	Round	Watery	Translucent
TUTMGSA126	South Africa	Nelspruit	102		Funsi	6	1	Round	Cream	Opaque
TUTMGSA150	South Africa	Nelspruit	103		Dowie	6	2	Oval	Watery	Translucent
TUTMGSA143	South Africa	Nelspruit	104		Sigiri	6	<1	Round	Watery	Translucent
TUTMGSA133	South Africa	Nelspruit	105		Dowie	5	2	Oval	Watery	Translucent
TUTMGSA106	South Africa	Nelspruit	106		Puffeun	4	2	Convex	Cream	Opaque
TUTMGSA135	South Africa	Nelspruit	106	R	Sigiri	6	<1	Round	Watery	Translucent
TUTMGSA142	South Africa	Nelspruit	107		Sigiri	6	2	Round	Watery	Translucent
TUTMGSA139	South Africa	Nelspruit	108		Puffeun	6	<1	Round	Watery	Translucent
TUTMGSA144	South Africa	Nelspruit	108		Sigiri	6	2	Oval	Watery	Translucent
TUTMGSA140	South Africa	Nelspruit	108		Funsi	6	<1	Round	Watery	Translucent
TUTMGSA132	South Africa	Nelspruit	109	S	Dowie	2	2	Convex	Watery	Translucent
TUTMGMZQ195	Mozambique	Muriaze	110		Belane M	5	4	Convex	Cream	Opaque
TUTMGGH84	Ghana	Damongo	111	T	Funsi	6	<1	Round	Cream	Opaque
TUTMGGH34	Ghana	Nyankpala[2]	112		Sigiri	6	1	Round	Pink	Opaque
TUTMGGH87	Ghana	Damongo	113		Funsi	6	<1	Round	Cream	Opaque
TUTMGGH89	Ghana	Damongo	113		Funsi	6	<1	Round	Cream	Opaque
TUTMGMZQ205	Mozambique	Muriaze	114		Boli	6	2	Round	Watery	Translucent
TUTMGMZQ199	Mozambique	Muriaze	115		Sigiri	6	<1	Round	Cream	Opaque
TUTMGMZQ200	Mozambique	Muriaze	115	U	Sigiri	9	2	Round	Watery	Translucent
TUTMGMZQ201	Mozambique	Muriaze	115		Boli	9	2	Round	Cream	Opaque
TUTMGMZQ202	Mozambique	Muriaze	115		Boli	6	1	Round	Cream	Opaque
TUTMGMZQ198	Mozambique	Muriaze	116		Belane M	7	1	Round	Cream	Opaque
TUTMGMZQ196	Mozambique	Muriaze	117		Belane M	9	1	Round	Cream	Opaque
TUTMGMZQ197	Mozambique	Muriaze	118		Belane M	9	1	Round	Cream	Opaque
TUTMGGH81	Ghana	Damongo	119		Dowie	6	1	Round	Cream	Opaque
TUTMGGH82	Ghana	Damongo	119	V	Dowie	2	<1	Round	Yellow	Opaque
TUTMGSA115	South Africa	Nelspruit	120		Boli	3	3	Convex	Watery	Translucent
TUTMGSA114	South Africa	Nelspruit	121		Boli	6	4	Irregular	Cream	Opaque
TUTMGSA118	South Africa	Nelspruit	121		Boli	6	1	Round	Watery	Translucent
TUTMGSA122	South Africa	Nelspruit	122		Funsi	6	2	Convex	Watery	Translucent
TUTMGSA105	South Africa	Nelspruit	123		Puffeun	6	2	Round	Watery	Translucent
TUTMGSA120	South Africa	Nelspruit	123		Boli	9	1	Round	Cream	Opaque
TUTMGSA109	South Africa	Nelspruit	124		Boli	6	4	Irregular	Watery	Translucent
TUTMGSA110	South Africa	Nelspruit	124		Boli	4	<1	Round	Watery	Translucent
TUTMGSA112	South Africa	Nelspruit	124	W	Boli	3	3	Irregular	Watery	Translucent
TUTMGSA119	South Africa	Nelspruit	124		Boli	6	2	Irregular	Watery	Translucent
TUTMGSA107	South Africa	Nelspruit	125		Funsi	8	1	Round	Watery	Translucent
TUTMGSA108	South Africa	Nelspruit	126		Boli	6	3	Round	Watery	Translucent
TUTMGSA145	South Africa	Nelspruit	127		Dowie	6	2	Oval	Watery	Translucent
TUTMGGH88	Ghana	Damongo	128		Funsi	6	<1	Round	Watery	Translucent
TUTMGGH91	Ghana	Damongo	129		Sigiri	5	<1	Round	Watery	Translucent
TUTMGGH92	Ghana	Damongo	130		Sigiri	6	<1	Round	Watery	Translucent

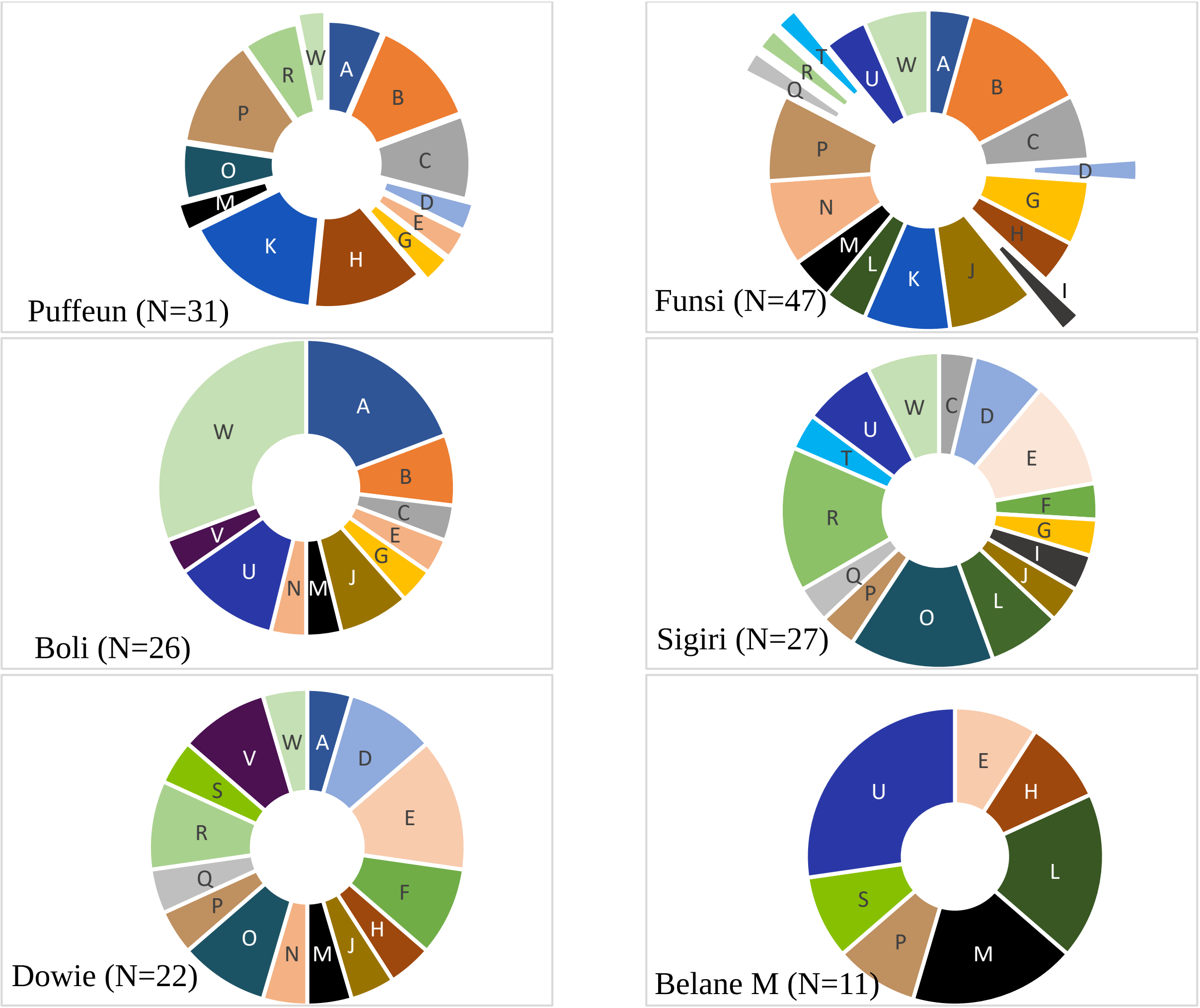

Fig. S1 The distribution of isolates (irrespective of the country of origin) from each Kersting's groundnut landrace within Box-PCR clusters. Uppercase letters indicate labels of major clusters as shown in Fig. 1. The area of each segment is proportional to the number of isolates occupying the cluster. N = Total number of isolates obtained from the respective landraces.

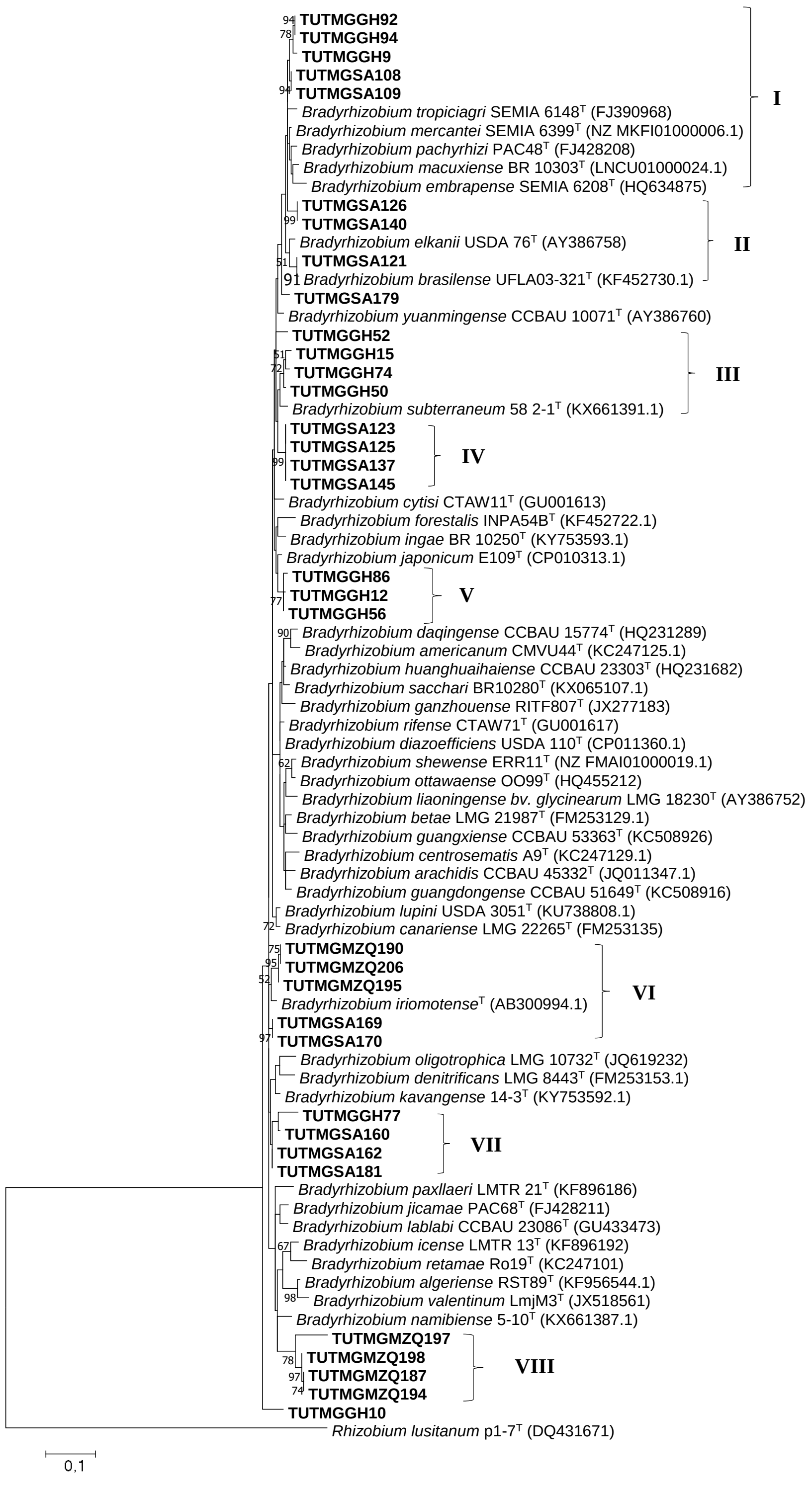

Fig. S2 Maximum likelihood molecular phylogenetic analysis of Kersting's groundnut isolates from Ghana, South Africa and Mozambique based on sequences of *atp*D gene. The analysis involved 77 nucleotide sequences. Roman numerals indicate the labels assigned to each cluster.

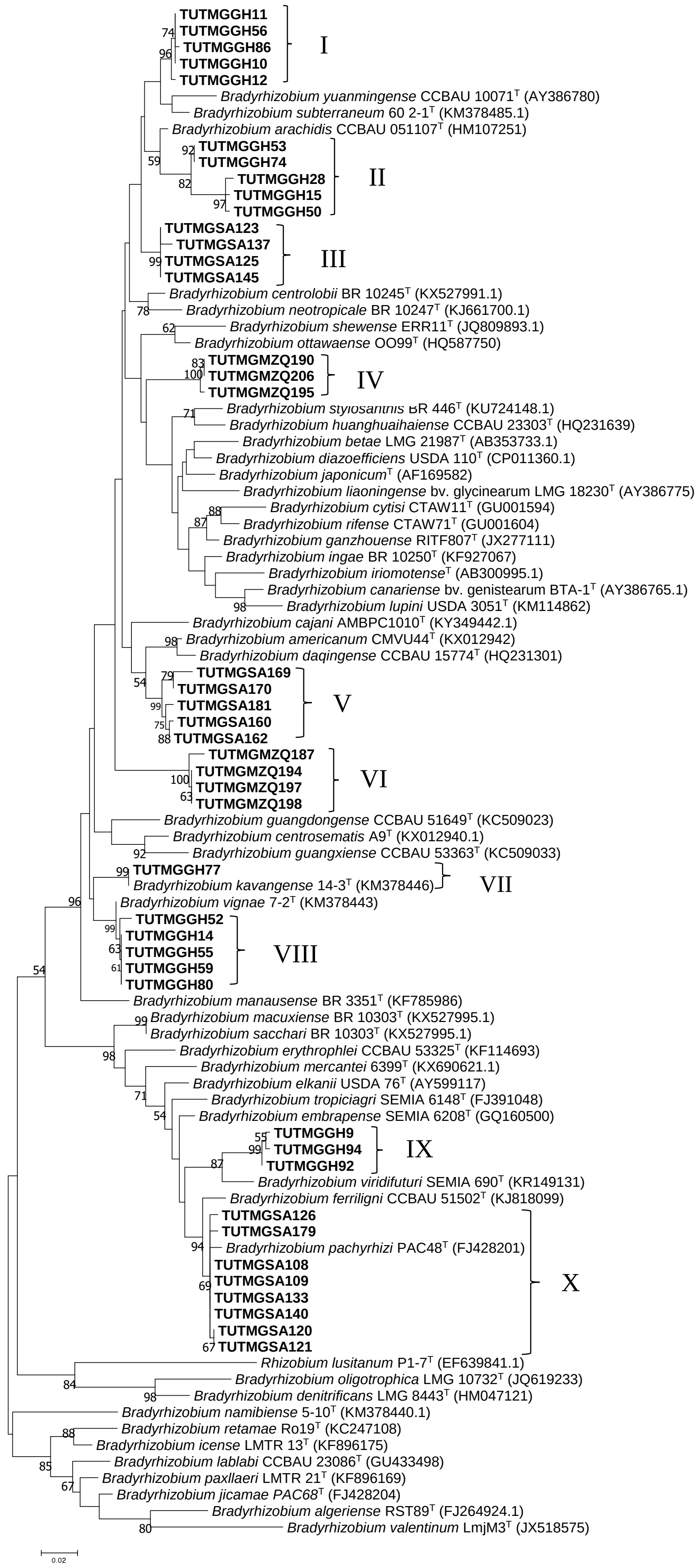

Fig. S3 Maximum likelihood molecular phylogenetic analysis of Kersting's groundnut isolates from Ghana, South Africa and Mozambique based on sequences of *gln*II gene. The analysis involved 93 nucleotide sequences. Roman numerals indicate the labels assigned to each cluster.

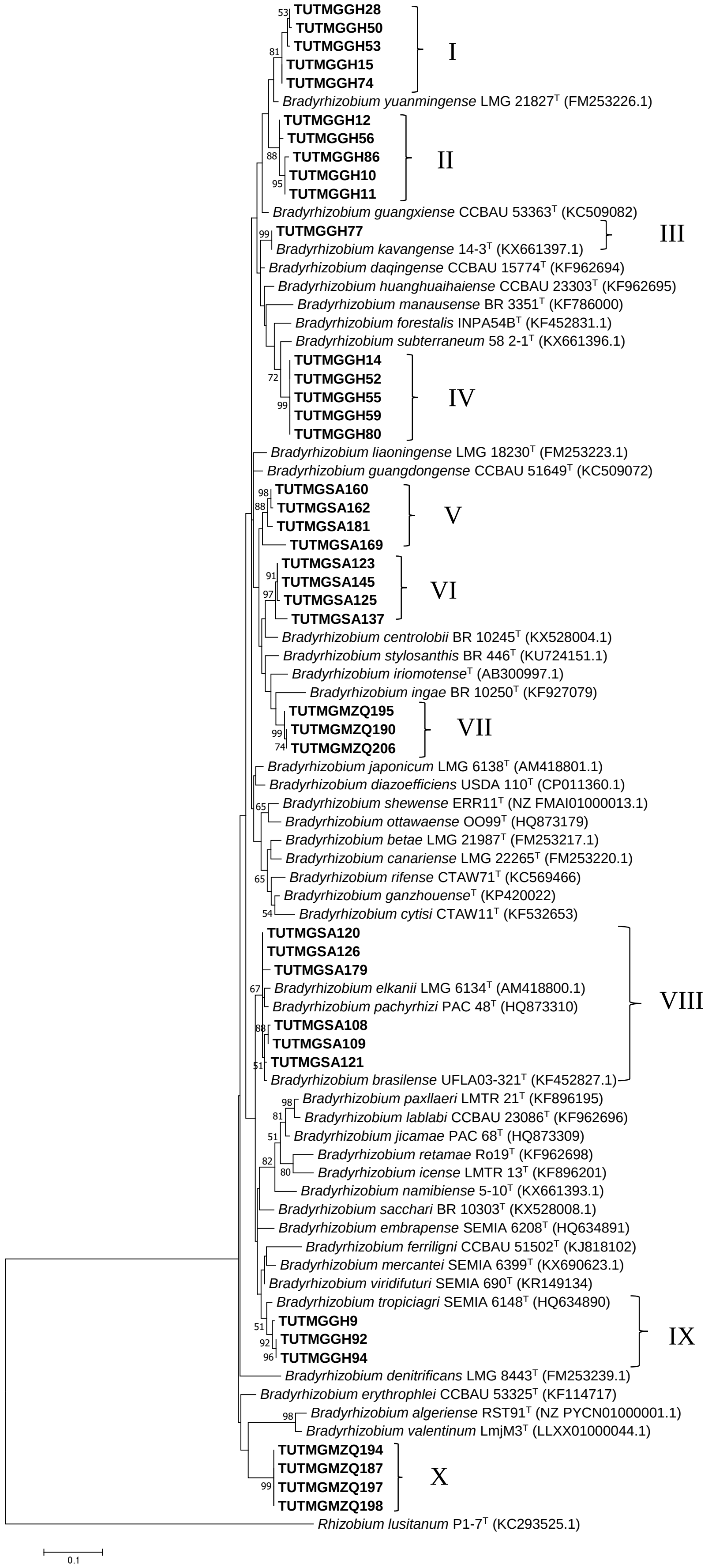

Fig. S4 Maximum likelihood molecular phylogenetic analysis of Kersting's groundnut isolates from Ghana, South Africa and Mozambique based on sequences of *gyr*B gene. The analysis involved 83 nucleotide sequences. Roman numerals indicate the labels assigned to each cluster.

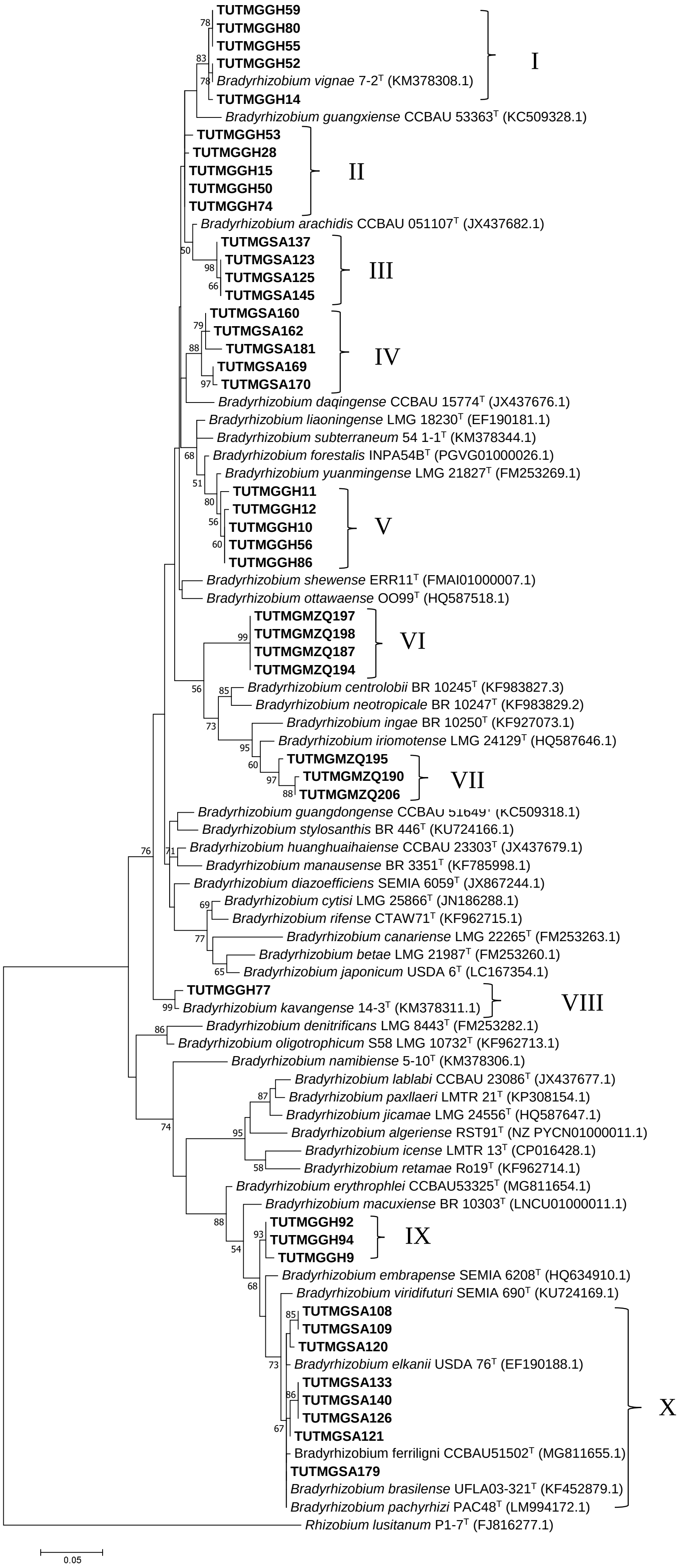

Fig. S5 Maximum likelihood molecular phylogenetic analysis of Kersting's groundnut isolates from Ghana, South Africa and Mozambique based on sequences of *rpo*B gene. The analysis involved 86 nucleotide sequences. Roman numerals indicate the labels assigned to each cluster.

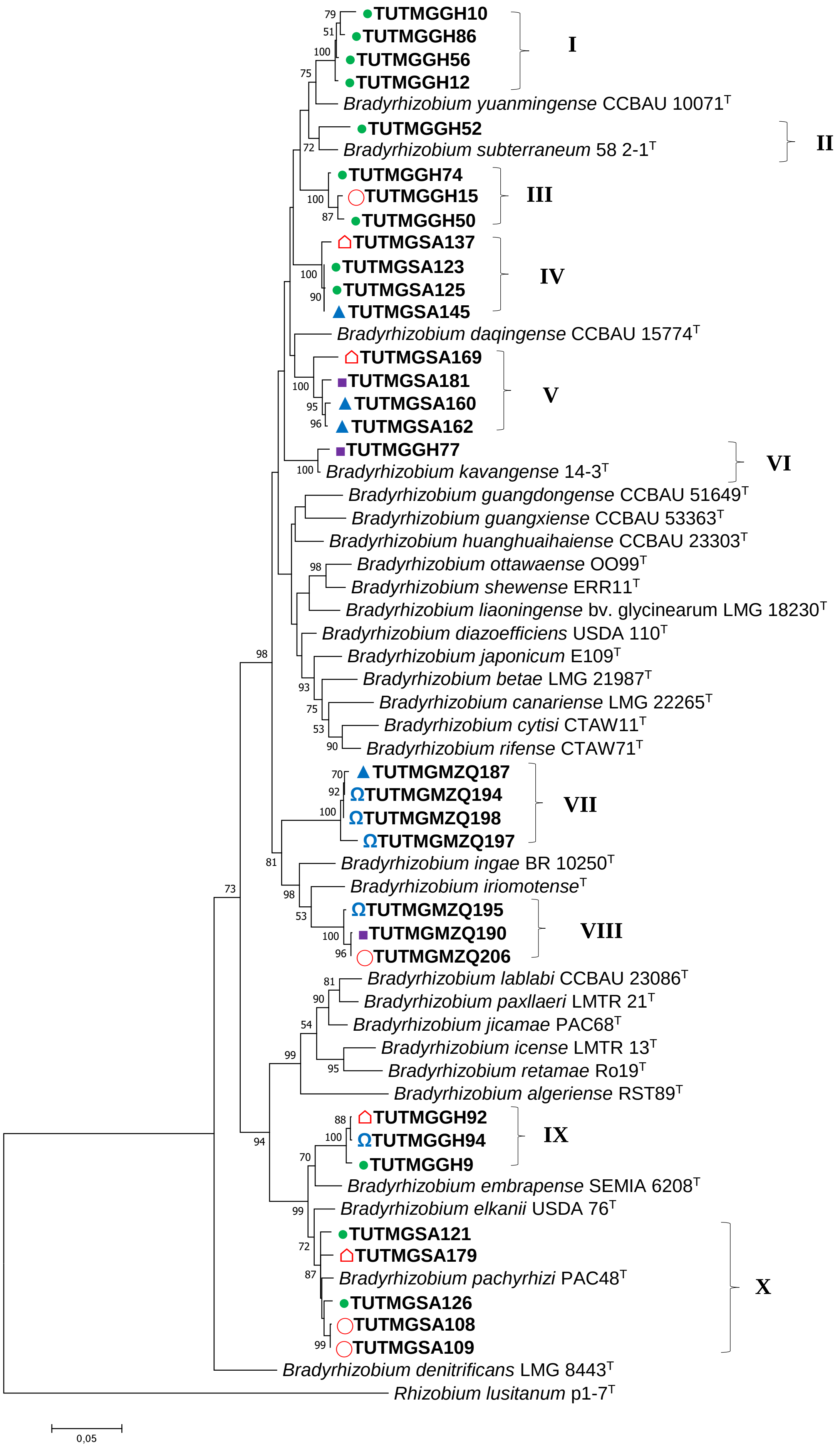

Fig. S6 Maximum likelihood phylogenetic analysis of Kersting's groundnut rhizobial isolates based on concatenated sequences of *atp*D-*gln*II-*gyr*B-*rpo*B genes. The analysis involved 61 nucleotide sequences. Roman numerals indicate the labels assigned to each cluster. For each isolate, the host landrace is indicated by colour coded symbols: ■Puffeun, ○Boli, ▲Dowie, •Funsi, ⌂Sigiri and ΩBelane

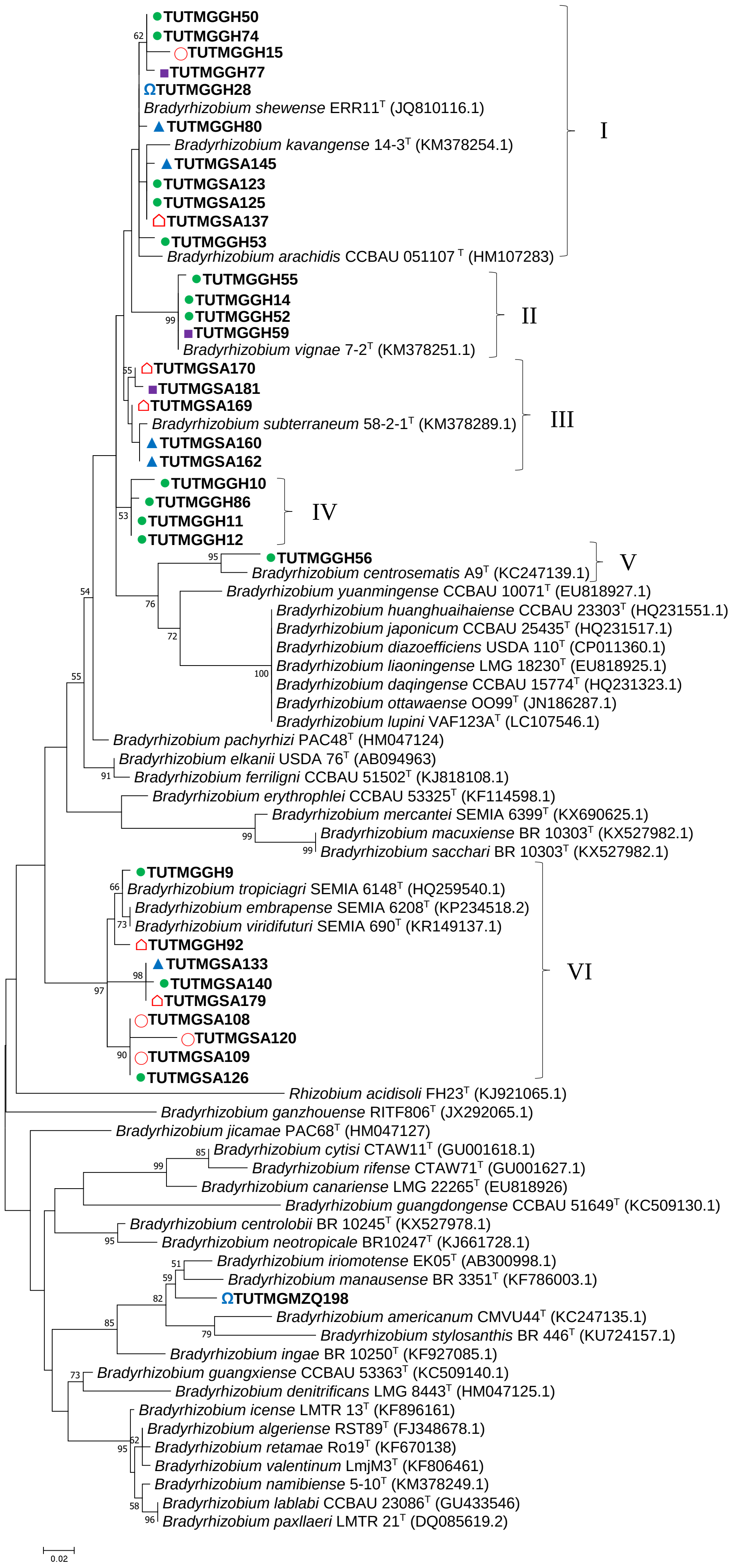

Fig. S7 Maximum likelihood molecular phylogenetic analysis of Kersting's groundnut isolates from Ghana, South Africa and Mozambique based on sequences of *nif*H gene. The analysis involved 82 nucleotide sequences. Roman numerals indicate the labels assigned to each cluster. For each isolate, the host landrace is indicated by colour coded symbols: ■Puffeun, ○Boli, ▲Blue, ●Funsi, ⌂Sigiri and ΩBelane mottled.
